# Diagnosis of Cognitive and Mental Disorders: A New Approach Based on Spectral–Spatiotemporal Analysis and Local Graph Structures of Electroencephalogram Signals

**DOI:** 10.3390/brainsci15010068

**Published:** 2025-01-14

**Authors:** Arezoo Sanati Fahandari, Sara Moshiryan, Ateke Goshvarpour

**Affiliations:** 1 Department of Biomedical Engineering, Imam Reza International University, Mashhad 91388-3186, Iran; arezo.sanati.2022@gmail.com (A.S.F.); sara.moshiryan1599@gmail.com (S.M.); 2 Health Technology Research Center, Imam Reza International University, Mashhad 91388-3186, Iran

**Keywords:** Alzheimer’s disease, depression, mild cognitive impairment, schizophrenia, Granger causality, local graph structures, electroencephalogram, spectral analysis, classification

## Abstract

Background/Objectives: The classification of psychological disorders has gained significant importance due to recent advancements in signal processing techniques. Traditionally, research in this domain has focused primarily on binary classifications of disorders. This study aims to classify five distinct states, including one control group and four categories of psychological disorders. Methods: Our investigation will utilize algorithms based on Granger causality and local graph structures to improve classification accuracy. Feature extraction from connectivity matrices was performed using local structure graphs. The extracted features were subsequently classified employing K-Nearest Neighbors (KNN), Support Vector Machine (SVM), AdaBoost, and Naïve Bayes classifiers. Results: The KNN classifier demonstrated the highest accuracy in the gamma band for the depression category, achieving an accuracy of 89.36%, a sensitivity of 89.57%, an F1 score of 94.30%, and a precision of 99.90%. Furthermore, the SVM classifier surpassed the other machine learning algorithms when all features were integrated, attaining an accuracy of 89.06%, a sensitivity of 88.97%, an F1 score of 94.16%, and a precision of 100% for the discrimination of depression in the gamma band. Conclusions: The proposed methodology provides a novel approach for analyzing EEG signals and holds potential applications in the classification of psychological disorders.

## 1. Introduction

Neurological and cognitive disorders, including Alzheimer’s disease, depression, mild cognitive impairment (MCI), and schizophrenia, pose significant challenges to global health, affecting millions of individuals worldwide [1,2]. The prevalence of these conditions is increasing, influenced by factors such as aging populations and socioeconomic stressors. Early and accurate diagnosis is crucial, as timely intervention can markedly enhance treatment outcomes, improve quality of life for affected individuals, and reduce the burden on healthcare systems. For instance, the early identification of MCI may lead to the implementation of strategies that delay its progression to more severe forms of dementia. Similarly, early intervention in the management of depression can help mitigate long-term social care and healthcare costs [3]. Furthermore, the early detection of schizophrenia can facilitate the formulation of effective treatment plans, minimize critical risks, and enhance long-term prognoses. Consequently, the advancement of diagnostic systems in this domain is of paramount importance.

The prompt and precise diagnosis of disorders is essential for effective treatment and management. However, the intricate and multifaceted characteristics of these disorders [4] present considerable challenges in clinical diagnosis. Conventional diagnostic methods, which predominantly depend on clinical interviews and behavioral assessments, are frequently subjective [4] and may fail to sufficiently elucidate the underlying neural mechanisms associated with the disorder. This inadequacy can result in delayed or inaccurate diagnoses. Consequently, there is a growing necessity for objective and quantitative diagnostic approaches that can offer more profound insights into brain function [5].

Recent advancements in neuroimaging techniques have heightened interest in the application of electroencephalogram (EEG) signals for the diagnosis of neurological disorders [6]. EEG serves as a non-invasive and cost-effective modality that measures electrical activity in the brain, thereby providing valuable insights into both normative and pathological brain functions. Although traditional EEG analysis predominantly emphasizes spectral characteristics [7,8], recent studies [9] suggest that the integration of spatial and temporal information can facilitate a more comprehensive understanding of the brain’s dynamic processes, thereby uncovering intricate patterns associated with various neurological disorders.

The analysis of frequency bands has emerged as a critical element in the processing of EEG signals for the diagnosis of various neurological disorders [10]. Each disorder typically manifests distinct alterations in specific frequency bands, which can be correlated with underlying pathophysiological mechanisms. For example, in Alzheimer’s disease, heightened activity in the gamma band during cognitive tasks has been linked to abnormal neuronal synchronization and network dysfunction [10]. Similarly, other disorders exhibit unique frequency-related patterns. In major depressive disorder (MDD), a reduction in alpha band activity, particularly in the frontal regions, has been noted, potentially indicating an impaired regulation of emotional and cognitive processes. Conversely, anxiety disorders are frequently associated with increased beta band activity in the frontal and central regions, which may reflect heightened neural arousal and hyperactivity [11]. Epilepsy is characterized by atypical patterns in the delta (0.5–4 Hz) and theta (4–8 Hz) bands; these lower frequencies often predominate during epileptic seizures, suggesting synchronized abnormal neural firing across various brain regions [12]. Attention-deficit/hyperactivity disorder (ADHD) is typically marked by an elevated theta-to-beta ratio, characterized by increased theta activity and decreased beta activity, which may indicate under-activation of the prefrontal cortex and challenges in attention regulation [13]. Sleep disorders, such as insomnia and obstructive sleep apnea, also exhibit distinct EEG patterns, including disruptions in the delta band during deep sleep and alterations in sleep spindles (12–16 Hz) [14]. This highlights the importance of frequency-domain features in identifying disease-specific patterns and elucidating the functional changes occurring within the brain. Therefore, the careful selection of appropriate frequency bands for analysis can significantly improve the accuracy and interpretability of diagnostic models.

Numerous studies have concentrated on the interpretation of EEG signals and the development of intelligent systems. These endeavors typically involve feature extraction methodologies [15] and machine learning techniques [16,17,18], with recent advancements integrating deep learning approaches. Significant challenges within the realm of machine learning include the necessity for a substantial volume of samples and the considerable computational resources required for the accurate diagnosis of multiple diseases. In contrast, various feature extraction techniques have been explored, including time-domain methods [19], frequency-domain approaches [15,16,17,20], non-linear features [15,21,22], and techniques based on brain connectivity [15,18,23,24].

Traditionally, EEG analysis methods have approached the data from each channel independently, concentrating on the characteristics of individual signals. However, there is increasing acknowledgment within the research community regarding the significance of investigating interactions among different brain regions [25,26], rather than analyzing each channel in isolation. A comprehensive understanding of the connectivity between various brain regions is essential for a thorough evaluation of neurological conditions [26]. The analysis of brain connectivity seeks to elucidate the interactions and dependencies among diverse brain areas, thereby providing deeper insights into the mechanisms underlying various disorders. Numerous methodologies have been explored for quantifying these connections [27,28], including Granger causality, correlation, and differential entropy. Each of these approaches generates a connectivity matrix, with rows and columns representing EEG electrodes, where each element quantifies the interaction between paired channels.

The precise quantification of connectivity measures is essential for providing reliable inputs to machine learning algorithms utilized in classification and prediction tasks [29]. To enhance the representation and analysis of connectivity data, advanced techniques, such as image processing algorithms, can be applied to connectivity matrices, thereby enabling their interpretation as graphs. This approach facilitates a more comprehensive examination of network properties and assists in the identification of patterns associated with various neurological conditions. By conceptualizing these matrices as images, we can leverage image processing techniques to extract meaningful patterns, identify significant connections, and improve the performance of diagnostic models. Consequently, this study employs an algorithm grounded in image processing techniques to investigate local connectivity patterns among different brain channels.

The investigation of relationships among EEG channels is a well-established area of research. While methods such as Granger causality and other techniques for analyzing channel interactions are not novel, the recent literature has increasingly highlighted their significance in elucidating brain connectivity and its implications for neurological disorders [30]. The field of EEG analysis has undergone substantial evolution over the decades, particularly with the emergence of sophisticated computational techniques and machine learning methodologies. This study builds upon this historical context while introducing innovative methodologies that enhance the granularity and applicability of connectivity analysis in contemporary clinical environments. This research presents a novel diagnostic framework for the simultaneous classification of multiple cognitive and mental disorders utilizing advanced EEG techniques, thereby addressing critical limitations in existing methodologies. A distinctive advantage of our approach, in comparison to prior methodologies, is its capacity to concurrently analyze multiple cognitive and mental disorders while incorporating advanced techniques such as local graph structures (LGSs) for feature extraction, which enriches the data representation. Moreover, by integrating spectral–spatiotemporal analysis with graph-theory-based connectivity measures, our approach facilitates a multi-dimensional perspective that aids in the identification of unique connectivity patterns associated with specific disorders. The primary innovations and contributions of this work are delineated as follows: (1) In contrast to previous studies that concentrate on a single disorder [17,19,20,31,32], this framework is designed to analyze and classify multiple cognitive and mental disorders simultaneously. By leveraging EEG data from diverse conditions, this approach offers a broader diagnostic capability, thereby addressing a significant gap in the literature. (2) The utilization of local graph structures is crucial in the advanced feature extraction process from EEG signals. This methodology incorporates techniques such as Singular Value Decomposition (SVD), log energy entropy, and Shannon entropy, which facilitate the extraction of more informative features from the data. These features provide a richer representation of brain activity, which is essential for differentiating between various conditions. (3) The integration of spectral–spatiotemporal analysis with graph-theory-based connectivity measures provides a multi-dimensional perspective on brain function. This synthesis enables the identification of unique connectivity patterns associated with specific disorders, thereby advancing EEG-based diagnostic techniques. (4) The evaluation of the system’s performance across each frequency band serves as a critical component of this study. By assessing the proposed framework’s effectiveness across different frequency ranges, this study aims to yield a more nuanced understanding of the system’s diagnostic capabilities. (5) The employment of well-established machine learning classifiers, including K-Nearest Neighbors (KNN), Support Vector Machine (SVM), AdaBoost, and Naïve Bayes, establishes a benchmark for comparison. This ensures that the results are not only robust, but also comparable with existing diagnostic methods, thereby further reinforcing the contributions of this study.

The proposed framework offers a cohesive and comprehensive methodology that enhances the domain of EEG-based diagnostics by facilitating the concurrent classification of various cognitive and mental disorders. This study introduces a diagnostic instrument that has exhibited both acceptable and high performance in the identification of multiple disorders. By providing a more versatile and clinically relevant diagnostic solution, it substantially contributes to the prompt detection and management of cognitive and mental health disorders. Moreover, it lays the groundwork for future research focused on refining EEG analysis techniques to encompass an even wider array of neurological conditions.

The article is organized in the following manner: Section 2 outlines the methodology; Section 2.1 details the database; Section 2.2 addresses the preprocessing steps; Section 2.3 examines feature extraction; Section 2.4 explores classification; Section 3 presents the results; Section 4 offers a discussion; and Section 5 concludes the study.

## 2. Materials and Methods

**Figure 1:**
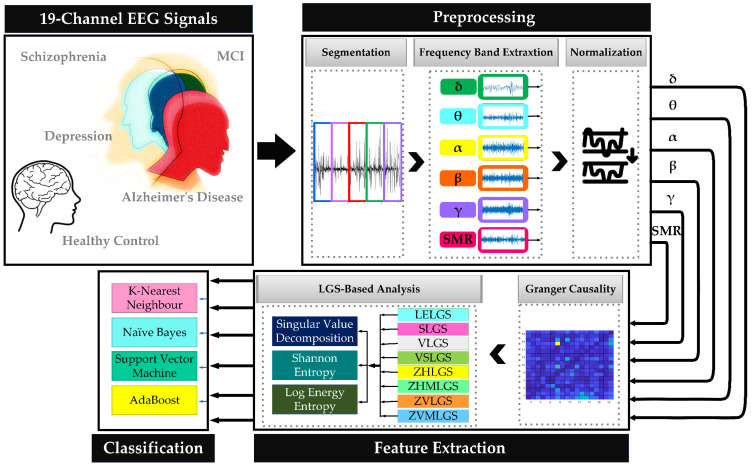
Block diagram of the proposed system.

The proposed framework initiates with the segmentation of signals and the extraction of EEG frequency bands. Following the normalization of the data, Granger causality is computed between each EEG electrode to construct the Granger matrix. Subsequently, eight LGS features are extracted from each Granger matrix, and the LGS attributes are quantified utilizing Singular Value Decomposition (SVD), log energy entropy, and Shannon entropy. Ultimately, four machine learning algorithms are evaluated for the classification of five states: healthy normal, schizophrenia, MCI, Alzheimer’s disease, and depression. Figure 1 presents a block diagram of the proposed scheme, with each stage of the process elaborated upon in the subsequent sections.

### 2.1. Database

This study employed a publicly accessible EEG dataset recorded by Benninger et al. [33]. The dataset included EEG recordings from 230 participants (average age of 58.2 ± 18.7 years with an age range of 18–91 years; 129 (56.1%) female), including 28 diagnosed with major depression (average age: 69.7 ± 14.8 years; range: 33–91 years; 20 (71.4%) female); 42 with schizophrenia (average age: 41.4 ± 16.8 years; range: 18–76 years; 15 (35.7%) female); 65 with cognitive impairment (average age: 72.9 ± 7.2 years; range: 60–87 years; 31 (47.7%) female), from which 25 (38.5%) were diagnosed with MCI (average age: 73.5 ± 6.0 years; range: 62–85 years; 11 (44%) female), and 40 with Alzheimer’s disease (average age: 72.6 ± 7.9 years; range: 60–87 years; 20 (50%) female). Additionally, 95 control individuals without neurological or psychiatric morbidity (average age: 52.2 ± 16.8 years; range: 19–80 years; 63 participants, or 66.3%, were female). Table 1 presents the demographic information regarding the participants in each group [33]. The EEG recordings were obtained retrospectively from the medical records of all participating patients. The EEG recordings were conducted in a standardized environment by a qualified technician. All participants underwent EEG sessions between 8 AM and 1 PM utilizing a Nihon Kohden (Nihon Kohden, Tokyo, Japan) surface EEG system (19-electrode standard as per the international 10–20 electrode placement system) with a sampling rate of 500 Hz. During the EEG recording sessions, participants were permitted to rest with their eyes both open and closed. It is noteworthy that participants who underwent sleep EEGs were excluded from the dataset to mitigate the confounding effects associated with sleep states. Individuals diagnosed with major depressive disorder (MDD) were hospitalized during the specified timeframe. This diagnosis was corroborated by two senior psychiatrists by the criteria delineated in the DSM-IV and DSM-V, following a psychiatric assessment that determined the severity of depression to be at least moderate. The diagnosis of schizophrenia was established by two senior psychiatrists based on the criteria outlined in the ICD-10. Participants with cognitive impairment were diagnosed with either MCI or Alzheimer’s disease by two senior neurologists, by the criteria established by the National Institute on Aging and the Alzheimer’s Association. Control participants consisted of individuals undergoing routine EEGs for reasons unrelated to neuropsychiatric conditions [33]. None of the participants in the control group had been diagnosed with any conditions that would classify them into the other groups. The exclusion criteria for this group included a diagnosis of bipolar disorder, substance abuse, psychiatric or general medical conditions that required hospitalization, a history of epilepsy or conditions necessitating the use of anticonvulsants, electroconvulsive therapy (ECT), vagus nerve stimulation, or transcranial magnetic stimulation (TMS). Furthermore, individuals with a history of traumatic brain injury or imaging findings suggestive of cerebrovascular diseases, including both ischemic and hemorrhagic stroke, were also excluded from participation.

### 2.2. Preprocessing

Efficient data preprocessing constitutes a critical phase that profoundly influences the quality and reliability of subsequent analyses. This section offers a thorough overview of the principal preprocessing techniques utilized in this study, which include segmentation, frequency band extraction, and normalization.

#### 2.2.1. Segmentation

The initial step in the data preprocessing pipeline involves the segmentation of raw data. This process entails dividing the continuous data stream into smaller, more manageable segments or windows. The selection of an appropriate segmentation strategy is critical, as it can significantly influence the extraction of relevant features and the overall performance of the analysis. In this study, due to the varying lengths of the data, we established the minimum threshold of channel signal data (166,000 samples). Subsequently, we partitioned the total length of the signal into five equal segments, each consisting of 30,000 samples, which corresponded to time intervals of 60 s, given a sampling rate of 500 Hz. This segmentation approach allowed us to effectively capture the temporal dynamics within the signals, as each segment represented a distinct portion of the overall waveform.

#### 2.2.2. Frequency Band Extraction

Following the segmentation of the data, the subsequent step entails the extraction of pertinent frequency bands. By concentrating on specific frequency bands, researchers can isolate and analyze the underlying patterns and rhythms in the data, which may yield significant insights. In this procedure, a Butterworth filter is applied to each segment to isolate six distinct frequency bands: delta (0.5–4 Hz), theta (4–8 Hz), alpha (8–13 Hz), beta (13–30 Hz), gamma (30–100 Hz), and Sensorimotor rhythm (SMR) (12–15 Hz) [15]. When shaping the frequency spectrum of a signal using a filter, the “transition band” of a basic first-order filter may become excessively long and wide, thereby necessitating the utilization of active filters with an order greater than one. For this research, a second-order Butterworth filter was employed to extract the frequency bands [34].

#### 2.2.3. Normalization

The final stage of the data preprocessing pipeline entails the normalization of the extracted features. Normalization is an essential procedure that ensures the data are uniformly scaled, thereby enabling meaningful comparisons and analyses. In this study, we utilized z-score normalization, transforming the data by subtracting the mean and dividing by the standard deviation. This standardization process ensures that all features possess a mean of zero and a standard deviation of one, effectively reducing the impact of the original scale and units of measurement (see Equation (1)).

z = (x − μ)/σ(1)

where μ symbolizes the population mean, while σ represents the population standard deviation. The absolute value of z reflects the distance between the raw score x and the population mean, expressed in standard deviation units. A negative z value indicates that the raw score is below the mean, whereas a positive z value signifies that it is positioned above the mean.

By utilizing these data preprocessing techniques, our aim is to prepare the data for subsequent analysis, thereby improving the reliability and interpretability of the results.

### 2.3. Feature Extraction

In the subsequent phase of the analytical process, we extract features from the EEG data, consisting of three primary components. Initially, we calculate Granger causality to evaluate the relationships among various channels of EEG signals. From this matrix, we derive eight features pertinent to the local graph structure (LGS) for further examination. Following the extraction of these features, we employ the Singular Value Decomposition (SVD) method to decompose the singular values, thereby acquiring a more nuanced representation structure of the EEG data. Additionally, we compute logarithmic energy entropy and Shannon entropy to assess the complexity of the EEG signal. These procedures are crucial for identifying the most effective predictive models for the analysis of EEG data.

#### 2.3.1. Granger Causality

Granger causality is a statistical concept utilized to assess the capacity of one time series to forecast another. Introduced by Clive Granger in the 1960s, this concept has since become a widely employed tool in econometrics and various other fields [35]. The foundational principle of Granger causality is predicated on the notion that if time series X causes time series Y, then the historical values of X should possess predictive power for the future values of Y. In essence, if X Granger causes Y, the information contained in X enhances the accuracy of predicting Y beyond what can be achieved by solely considering the past values of Y [35].

Consider two signals, x(t) and y(t). If x is determined to be the cause of y according to the Granger causality principle, then the historical values of x should provide valuable information for predicting the future values of y. In contrast, relying exclusively on the historical values of y is often insufficient for accurately forecasting its future values [36]. To perform the univariate autoregression of y(t) (as represented in Equation (2)), the optimal lagged values of y, denoted as y(t − i), are first computed. This initial process is subsequently enhanced by integrating the lagged values of x(t) (as shown in Equation (3)).

(2) $$y\left(t\right)=e\left(t\right)+\sum_{i=1}^{\infty }a\left(i\right)\cdot y\left(t-i\right)$$

(3) $$y(t)=ẽ(t)+\sum_{i=1}^{\infty }a\left(i\right)\cdot y\left(t-i\right)+\sum_{j=1}^{\infty }b\left(i\right)\cdot x\left(t-j\right)$$

In this context, a(i) and b(j) represent the regression coefficients, while e(t) and ẽ(t) denote the calculated prediction errors without and with the inclusion of lagged values of x(t) in the prediction of y(t), respectively. The variances of these errors are known as var(e) and var(ẽ). If var(ẽ) is smaller than var(e), then x(t) Granger causes y(t), indicating a Granger causality of 1. Conversely, if var(ẽ) is larger than var(e), this suggests x(t) does not Granger cause y(t), corresponding to a Granger causality of 0.

The application of Granger causality in the analysis of EEG signals significantly enhances our understanding of the interconnections among various brain regions and facilitates the detection of brain activity patterns elicited by diverse stimuli or tasks. Such insights contribute to a more comprehensive understanding of human cognitive, behavioral, and neurophysiological processes [35]. Granger causality is a valuable methodological tool for investigating the dynamics of brain activity and the interactions among different regions during cognitive tasks. By employing this analytical framework on EEG data, researchers can uncover causal relationships and the flow of information within the brain, illuminating the underlying neural processing mechanisms. Ultimately, this enhanced understanding of brain connectivity and function has the potential to drive advancements in neuroscience, psychology, and medicine [35].

#### 2.3.2. LGS-Based Analysis

The local graph structure (LGS) is a computationally efficient operator employed for the extraction of local features from a matrix. In the present study, rather than applying this method directly to the matrix, we utilized it to analyze the 19 × 19 Granger matrix comparably.

Before extracting local features, matrix I, with dimensions m × n, is partitioned into smaller sub-regions with dimensions m′ × n′, where m′ ≪ m and n′ ≪ n. Subsequently, the LGS operator is applied to each sub-region, computing a transformed value derived from a directed local graph structure established by the neighboring cells.

**Figure 2:**
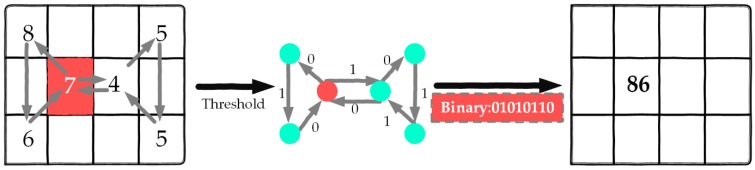
Local graph structure. Decimal calculation steps: (01010110)_2_ = (0 × 2^7^) + (1 × 2^6^) + (0 × 2^5^) + (1 × 2^4^) + (0 × 2^3^) + (1 × 2^2^) + (1 × 2^1^) + (0 × 2^0^).

The neighboring cells are assessed in relation to the source cell by the graph direction. When labeling the edges of the local graph for a cell, the value of the source cell is compared to that of its neighboring cells to calculate the differences. If the difference is greater than or equal to zero, a value of 1 is assigned to the edge; conversely, a value of 0 is assigned if the difference is less than zero. Subsequently, the binary values (0 s and 1 s) from the edges of the directed local graph are concatenated following the graph’s direction, resulting in an 8-bit binary number. This binary number is then converted into a decimal number, which is assigned to the corresponding target cell. Each cell is associated with a unique decimal number generated by using the directed LGS, along with its corresponding binary number, as demonstrated in Figure 2 [37].

This study employed eight distinct local graph structures (LGSs) [37], which include the logically extended local graph structure (LELGS), symmetric local graph structure (SLGS), vertical local graph structure (VLGS), vertical symmetric local graph structure (VSLGS), zigzag horizontal local graph structure (ZHLGS), zigzag horizontal middle local graph structure (ZHMLGS), zigzag vertical local graph structure (ZVLGS), and zigzag vertical middle local graph structure (ZVMLGS).

The symmetric local graph structure (SLGS) was developed to improve matrix texture by extracting texture information from neighboring pixels in a balanced manner. In contrast to the conventional local graph structure (LGS), which relies on four neighboring pixels, SLGS incorporates seven neighboring pixels in conjunction with the target cell as a threshold. This methodology facilitates a more equitable extraction of texture information from both the left and right sides of the target pixel.

**Figure 3:**
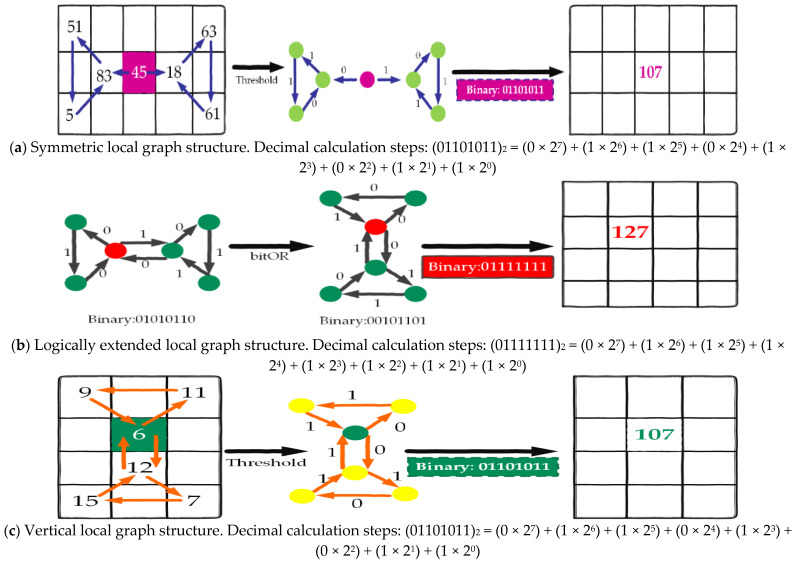

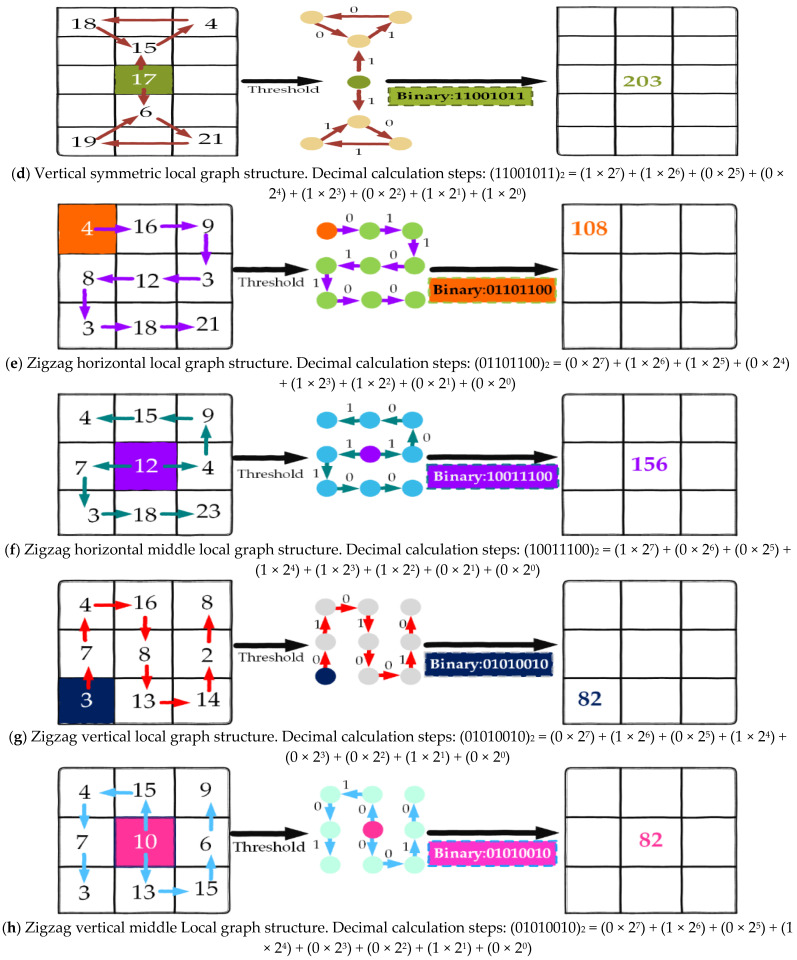
An illustration of the eight distinct LGSs utilized in the present study. (**a**) Symmetric local graph structure; (**b**) Logically extended local graph structure; (**c**) Vertical local graph structure; (**d**) Vertical symmetric local graph structure; (**e**) Zigzag horizontal local graph structure; (**f**) Zigzag horizontal middle local graph structure; (**g**) Zigzag vertical local graph structure; (**h**) Zigzag vertical middle Local graph structure.

In the SLGS process, the Granger causality matrix is partitioned into multiple blocks, each consisting of 3 × 5 cells. The graph edges within the SLGS are labeled beginning at the target pixel and progressing counterclockwise to the left region, resulting in a 4-bit binary string. Subsequently, labeling continues from the target pixel, advancing clockwise to the right region, generating an additional 4-bit binary string. The concatenation of these two strings yields an 8-bit binary pattern corresponding to the target pixel. The edge labeling methodology employed in the LGS is similarly applied in the SLGS to produce binary edge labels, which are converted into decimal values. Figure 3a provides a visual representation of the SLGS graph structure for each pixel.

The LELGS, as introduced by Rakshid et al. [37], utilizes a 4 × 4 overlapping block, a signum function, and a bitwise OR operator to extract 8-bit binary features. This methodology significantly depends on the 4 × 4 overlapping block for comprehensive feature extraction. By integrating the signum function with the bitwise OR operator, the LELGS effectively captures complex patterns and relationships within the data. The incorporation of vertical and horizontal graphs further enhances feature extraction, facilitating the derivation of 8-bit binary features that encapsulate critical information. Figure 3b presents a graphical representation of the LELGS approach, elucidating its innovative mechanisms.

The vertical local graph structure (VLGS), an advanced iteration of the LGS, also introduced by Rakshid et al. [37], employs a 4 × 3 overlapping block configuration. This descriptor is characterized by its utilization of a vertical graph, which is why it is named VLGS. Figure 3c provides a graphical representation of the VLGS. By leveraging the vertical graph structure, the VLGS captures both local and global information. The integration of a 4 × 3 overlapping block significantly enhances the descriptor’s robustness against variations in scale and orientation. Rakshid et al. [37] demonstrated the efficacy of the VLGS across a range of computer vision tasks, highlighting its superior accuracy and efficiency compared to traditional descriptors. The visual representation in Figure 3c underscores the distinctive characteristics of the descriptor.

The vertical symmetric local graph structure (VSLGS), which serves as the symmetric model of the VLGS, was introduced by Rakshid et al. [37]. This model employs overlapping blocks of size 5 × 3 in conjunction with the signum function for feature extraction. Figure 3d presents a numerical example that elucidates the operational mechanics of the VSLGS. The primary objective of this model is to enhance the performance of the VLGS by integrating symmetrical properties. The incorporation of 5 × 3 overlapping blocks alongside the signum function facilitates a more comprehensive approach to feature extraction, thereby yielding improved accuracy across a range of tasks. The graphical representation provided in Figure 3d further clarifies the functioning of the VSLGS, offering a specific example that aids in the better understanding and practical application of the model in real-world contexts.

The zigzag horizontal local graph structure (ZHLGS), which employs a horizontally aligned neighborhood block of dimensions 3 × 3, was introduced by Rakshid et al. [38]. An illustrative example of the ZHLGS is presented in Figure 3e.

In the ZHMLGS (zigzag horizontal middle local graph structure), the matrix is partitioned into blocks of size 3 × 3, and a specific pattern, in conjunction with the signum function, is employed for feature extraction. The utilized pattern is a zigzag horizontal middle graph. A graphical representation of the ZHMLGS is illustrated in Figure 3f [38]. This pattern is meticulously designed to capture the spatial information within each matrix block. By implementing the zigzag horizontal middle graph, it becomes possible to extract distinctive features that can be utilized for subsequent analysis and classification tasks. This methodology presents a novel approach to feature extraction, aimed at improving the overall efficacy of matrix processing algorithms.

The ZVLGS (zigzag vertical local graph structure) aims to extract vertical features from a matrix through the application of a zigzag pattern. This process is achieved by employing overlapping blocks, each measuring 3 × 3, as illustrated in Figure 3g [38].

The ZVMLGS (zigzag vertical middle local graph structure) represents an adaptation of the ZVLGS methodology. In the ZVMLGS framework, the central cell within the block is designated as the initial reference point. Figure 3h demonstrates the application of the zigzag vertical middle graph for feature extraction [38].

After calculating the features for each item across various bands, we proceed with the computations of Singular Value Decomposition, Shannon entropy, and logarithmic energy entropy.

- Singular Value Decomposition

Singular Value Decomposition (SVD) is a significant technique utilized in both computer science and mathematics for the decomposition of a matrix into specific coefficients. This process entails the breakdown of a matrix into three smaller rectangular matrices: the U matrix, the Σ matrix, and the V matrix. These matrices correspond to the left singular vectors, the diagonal singular values, and the right singular vectors, respectively. The Σ matrix contains the singular values of the original matrix, organized in descending order. By reducing the dimensions of the Σ matrix, it is possible to effectively preserve essential information from the original matrix while optimizing its dimensions. Specifically, the Singular Value Decomposition of an *m* × *n* complex matrix M can be expressed in the following factorization form (Equation (4)) [39]:

(4) $$X=U\Sigma V^{*}$$

In this context, let U represent an *m* × *m* complex unitary matrix, Σ denote an *m* × *n* rectangular diagonal matrix containing non-negative real numbers along its diagonal, and V signify an *n* × n complex unitary matrix. V^*^ is the conjugate transpose of V. This decomposition is universally applicable to any complex matrix. In instances where M is a real matrix, both U and V are real orthogonal matrices. In such scenarios, the SVD is typically denoted by UΣV^T^.

- Shannon Entropy

Shannon entropy is a fundamental concept within information and communication theory, first introduced by Engineer Earl William Shannon in the 1940s [40]. It serves as a measure of the complexity of a system, reflecting its inconsistency and incompleteness. Shannon entropy was chosen for its capacity to effectively quantify the uncertainty or unpredictability of information in a data-driven manner, thereby obviating the necessity for pre-established assumptions and models [41]. A higher entropy value indicates increased uncertainty and complexity, a characteristic of more diverse and unpredictable EEG patterns. Conversely, a lower entropy value signifies a decrease in uncertainty, suggesting a more regular and predictable set of EEG patterns.

While various entropy measures provide alternative perspectives on complexity, we have prioritized Shannon entropy due to its well-established theoretical foundation and extensive applicability as a complexity measure in neuroscience [42]. Furthermore, the simplicity of Shannon entropy enables easier integration with our other feature extraction techniques, facilitating a comprehensive analysis of EEG signals.

The Shannon entropy of a system can be calculated using the formula presented in Equation (5) [40]:

(5) $$\;H(X)=-\Sigma \;p(x)\;*\;{log}_{2}(p(x))$$

In this equation, H(X) signifies the system entropy, p(x) indicates the probability associated with each state of the system, and log2 denotes the logarithm to base 2. This calculation enables the evaluation of system complexity, thereby enhancing performance and efficiency [43]. Shannon’s entropy is instrumental in various applications, including information compression, data encoding, and algorithm optimization. It is a robust tool for problem solving and system improvement within information and communication theory.

- Logarithmic Energy Entropy

The entropy associated with the logarithm of energy represents a fundamental concept in both physics and information theory, enabling the quantification of a system’s complexity. This concept finds diverse applications in physics, cryptography, and communication, thereby aiding in the formulation of optimal algorithms for problem solving. The calculation of the entropy of the logarithm of a system’s energy is performed using the following formula (Equation (6)) [43]:

(6) H(X)=−Σ p(x) ∗ ln(p(x))

In this formula, H(X) denotes the entropy of system X, while p(x) represents the probability of each state within the system [43]. The logarithmic energy entropy facilitates the quantification of the information required to characterize a system, thereby enhancing its efficiency and performance. This concept is of considerable significance in both fundamental and applied research, contributing to the advancement of various technologies.

Once the SVD, log energy entropy, and Shannon entropy have been calculated for each LGS feature, the classifier is utilized. It is trained on the extracted features and subsequently employed to predict new data points.

### 2.4. Classification

This study employed four well-established classification algorithms—KNN, SVM, AdaBoost, and Naïve Bayes—for data classification. The KNN algorithm operates by identifying the nearest neighbors of a given sample within the feature space, which are subsequently utilized for classification. Initially, the algorithm finds the K samples closest to the new sample in the feature space. The majority class among these K neighbors is then used to assign the class to the new sample. The parameter K is a critical component of this algorithm and must be specified by the user. Its value significantly influences the classification performance. It is essential to select an appropriate value for K to achieve optimal results with the K-Nearest Neighbors algorithm. A small value of K may introduce noise into the classification process, whereas a large value can result in over-smoothing. Consequently, after evaluating various values of K, we determined that the optimal value is 5, which yielded the best classification results.

The Naïve Bayes algorithm is a probabilistic classification method that employs Bayes’ theorem and operates under the assumption of independence among features. This algorithm classifies new samples by calculating the conditional probability of each feature within each class. The feature independence assumption significantly simplifies the calculations associated with this algorithm, making it both straightforward and efficient. Despite its simplicity, Naïve Bayes consistently demonstrates robust performance and is well regarded for its rapid training speed and minimal computational requirements.

Support Vector Machines (SVMs) represent a supervised learning model that demonstrates proficiency in both linear and nonlinear classification tasks. This is achieved by constructing a hyperplane, or a collection of hyperplanes, within a high-dimensional space to effectively segregate data points into distinct classes. The algorithm’s objective is to maximize the margin, defined as the distance between the hyperplane and the nearest data points from any class, referred to as support vectors. This maximization contributes to establishing a robust decision boundary, enhancing the model’s generalization capabilities when applied to unseen data. A pivotal feature of SVMs is the kernel trick, which facilitates data mapping into a higher-dimensional space, thereby addressing complex nonlinear problems. In this study, the radial basis function (RBF) was employed as the kernel.

AdaBoost, or Adaptive Boosting, is an ensemble learning technique that integrates the predictions of multiple weak regressors, commonly decision trees, to construct a robust predictive model. This method is particularly effective in identifying complex relationships within data and is well suited for tasks that involve nonlinearity or interactions among features.

To evaluate the performance of algorithms, several criteria, including accuracy, sensitivity, precision, and F1 score, were employed [44]. These criteria facilitate a comprehensive assessment of classifier performance from multiple perspectives, allowing for a reliable and accurate comparison of results. Accuracy is defined as the ratio of correct predictions made by the model comparison to the total number of predictions, thereby indicating the percentage of instances in which the model has accurately forecasted outcomes (see Equation (7)) [44].

(7) $$Accuracy=\frac{True\;Positive+True\;Negative}{True\;Positive+True\;Negative+False\;Positive+False\;Negative}$$

While accuracy quantifies the overall proportion of correct predictions, precision specifically emphasizes the predictions related to the positive class. It is defined as the ratio of true positive predictions to the total number of positive predictions made by the model. The formula for calculating precision is presented in Equation (8) [44].

(8) $$Precision=\frac{True\;Positive}{True\;Positive+False\;Positive}$$

Sensitivity is defined as the ratio of true positive predictions to the total number of actual positive cases within the dataset, as represented in Equation (9) [44].

(9) $$Sensitivity=\frac{True\;Positives}{True\;Positive+False\;Negative}$$

The F1 score is a performance metric that integrates Precision and Recall into a single measure. It is computed as the harmonic mean of Precision and Recall, thereby providing a balanced assessment of a model’s performance (see Equation (10)) [44].

(10) $$F1\;score=2*\frac{Precision*Recall}{Precision+Recall}$$

In this study, the performance of machine learning models was assessed utilizing the 10-fold cross-validation technique in conjunction with the one-versus-all (OVA) classification method. The OVA technique serves as a strategy for training binary classifiers within multiclass classification problems, wherein each class is regarded as an independent binary classification task. Specifically, a binary classifier is developed for each class in the dataset, differentiating between instances of that class and instances belonging to all other classes. This methodology facilitates the simplification of the multiclass problem by transforming it into several binary classification tasks.

Performance metrics, including accuracy, sensitivity, precision, and specificity, were computed over ten iterations. The average values of these metrics were calculated and presented as the final evaluation of the machine learning models.

## 3. Results

Table 2 provides a detailed overview of the results obtained from the KNN classification model, which was applied to various features such as Shannon entropy, log energy entropy, and SVD, utilizing data from different EEG frequency bands. The rows in the table represent distinct proposed quantifiers, subdivided into five sub-rows that correspond to the five classes under classification. The dataset encompasses five classes: Class 1 for schizophrenia, Class 2 for mild cognitive impairment (MCI), Class 3 for depression, Class 4 represents controls (healthy individuals), and Class 5 signifies Alzheimer’s disease. The columns present the average values and standard deviations for the classification metrics, which include accuracy, sensitivity, F1 score, and precision.

The application of the KNN classifier to the delta band resulted in the highest classification accuracy for Class 3 when employing the log energy entropy feature, achieving an accuracy of 88.63%. In this instance, the sensitivity was recorded at 89.06%, the F1 score was 93.93%, and precision was perfect at 100%. Furthermore, KNN successfully classified Class 3 using the SVD and Shannon entropy features, with accuracies of 88.61% and 88.55%, respectively. These results are aligned with the accuracy obtained using log energy entropy for Class 3, indicating that the delta band is particularly effective for this classification task. For SVD, the sensitivity, F1 score, and precision values were 88.98%, 93.93%, and 99.90%, respectively. In contrast, for the Shannon entropy feature, the sensitivity, F1 score, and precision values were 88.94%, 93.89%, and 99.81%, respectively. Following Class 3, the next best performance was observed for Class 2, where the highest accuracy was achieved with the Shannon entropy feature, yielding values of 88.55% for accuracy, 89.01% for sensitivity, 93.90% for the F1 score, and 100% for precision. The recognition accuracy for Class 2 using log energy entropy and SVD was recorded at 88.46% and 87.97%, respectively. Conversely, the lowest recognition rate was observed for Class 4, with the lowest accuracy of 59.20% achieved using SVD.

The application of the KNN classifier to the theta band revealed that the highest classification accuracy for Class 3 was attained through the utilization of the log energy entropy feature, resulting in an accuracy of 89.29%. In this instance, the sensitivity was measured at 89.74%, the F1 score was 94.27%, and the precision was 100%. Additionally, KNN effectively identified Class 3 using the SVD and Shannon entropy features, achieving accuracies of 88.80% and 88.64%, respectively. These values are closely comparable to the accuracy obtained with log energy entropy for Class 3, suggesting that the theta band is particularly effective for this classification task. For SVD, the corresponding values for sensitivity, F1 score, and precision were 89.05%, 94.01%, and 99.90%, respectively. In the case of Shannon entropy, the sensitivity, F1 score, and precision were 89.04%, 93.94%, and 99.90%, respectively. Following Class 3, the next highest performance was observed for Class 2, where the maximum accuracy was achieved using Shannon entropy, yielding an accuracy of 88.45%, a sensitivity of 88.82%, an F1 score of 93.84%, and a precision of 99.81%. The recognition accuracy for Class 2 using log energy entropy and SVD was 88.36% for both methods. The minimum recognition rate was recorded for Class 4, with the lowest accuracy of 60.12% achieved through log energy entropy in the analysis of the alpha band employing the KNN classifier; the highest classification accuracy for Class 2 was attained with the log energy entropy feature, yielding an accuracy of 89.26%. The sensitivity for this classification was recorded at 89.47%, while the F1 score and precision were 94.25% and 100%, respectively. Additionally, the KNN classifier successfully identified Class 2 using SVD and Shannon entropy, achieving accuracies of 88.88% and 88.63%, respectively. These results are comparable to the accuracy obtained with log energy entropy for Class 2, indicating that the alpha band is particularly effective for this classification task. For the SVD method, the sensitivity, F1 score, and precision values were 89.25%, 94.06%, and 99.81%, respectively. In the case of Shannon entropy, the corresponding values were 89.03%, 93.92%, and 99.81%. Following Class 2, the next highest performance was observed for Class 3, where the maximum accuracy was achieved using Shannon entropy, resulting in an accuracy of 88.79%, a sensitivity of 89.25%, an F1 score of 94.01%, and a precision of 99.90%. The recognition accuracy for Class 3 using log energy entropy and SVD was 88.37% and 88.64%, respectively. The lowest recognition rate for the alpha band was noted for Class 4, with an accuracy of 58.69% achieved through the use of log energy entropy.

In the context of the SMR band, the KNN classifier demonstrated that the highest classification accuracy for Class 3 was achieved through the application of Shannon entropy, resulting in an accuracy of 89.05%. In this instance, the sensitivity was recorded at 89.22%, the F1 score was 94.15%, and the precision was 100%. Additionally, the KNN classifier effectively identified Class 3 using the SVD and log energy entropy features, achieving accuracies of 88.36% and 88.94%, respectively. These results are closely aligned with the accuracy obtained through Shannon entropy, suggesting that the SMR band is particularly effective for Class 3. For SVD, the sensitivity, F1 score, and precision values were 88.97%, 93.79%, and 99.71%, respectively. In the case of log energy entropy, the sensitivity, F1 score, and precision were 89.20%, 94.08%, and 99.90%, respectively. Following Class 3, the next highest performance was for Class 2, where the maximum accuracy was attained using SVD, yielding an accuracy of 88.48%, a sensitivity of 88.48%, an F1 score of 93.86%, and a precision of 100%. The recognition accuracy for Class 2 using log energy and Shannon entropy was recorded at 88.39% and 88.38%, respectively. The minimum recognition rate for the SMR band was encountered with Class 4, with the lowest accuracy of 57.35% achieved using SVD.

When the KNN classifier was applied to the beta band, the highest classification accuracy was attained with log energy entropy for Class 2, yielding an accuracy of 89.28%. The corresponding sensitivity was 89.55%, the F1 score was 94.26%, and the precision was 99.90%. The corresponding KNN classifier successfully identified Class 2 using SVD and Shannon entropy features, both of which achieved accuracies of 88.61%. These results are closely aligned with the accuracy obtained through log energy entropy, indicating that the beta band is particularly effective for Class 2. For the SVD method, the sensitivity, F1 score, and precision values were 89.28%, 93.89%, and 99.52%, respectively. In the case of Shannon entropy, the corresponding values were 89.11%, 93.93%, and 100%. Following 2, Class 3 exhibited the next best performance, with the highest accuracy also achieved using log energy entropy, resulting in an accuracy of 89.10%, a sensitivity of 89.14%, an F1 score of 94.18%, and a precision of 99.90%. The recognition accuracy for Class 3 using SVD and Shannon entropy was 88.80% and 88.65%, respectively. The lowest recognition rate for the beta band was observed for Class 4, with the lowest accuracy of 60.22% achieved using SVD.

In analyzing the gamma band using the KNN classifier, the highest classification accuracy was attained for Class 3, reaching an accuracy of 89.36% when employing SVD. In this context, the sensitivity was recorded at 89.57%, the F1 score at 94.30%, and the precision at 99.90%. The KNN classifier also effectively identified Class 3 using features derived from log energy entropy and Shannon entropy, achieving accuracies of 88.46% and 88.37%, respectively. These results are closely aligned with the accuracy obtained using SVD for Class 3, thereby highlighting the efficacy of the gamma band for this classification task. For log energy entropy, the sensitivity, F1 score, and precision were 89.04%, 93.83%, and 99.52%, respectively. In the case of Shannon entropy, the corresponding values were 89.20%, 93.78%, and 99.52%. Following Class 3, the next highest performance was observed for Class 2, where the maximum accuracy was achieved using SVD, with values of 88.87% for accuracy, 89.49% for sensitivity, 94.02% for F1 score, and 99.52% for precision. The recognition accuracy for Class 2 using log energy and Shannon entropy was recorded at 88.54% for both features. The lowest recognition rate for the gamma band was noted for Class 4, with the minimum accuracy using log energy entropy at 63.60%.

The results presented in Table 2 elucidate the efficacy of KNN classification, employing electroencephalogram (EEG) data across various frequency bands and feature extraction methodologies. Notably, Class 2, representing mild cognitive impairment, consistently exhibits the highest levels of accuracy, sensitivity, F1 score, and precision across all frequency bands and feature extraction techniques. Class 3, which corresponds to depression, also demonstrates commendable performance, albeit with slightly less consistency. Conversely, Class 4, representing controls, consistently achieves lower scores across all metrics, indicating difficulties in differentiating healthy individuals from those with cognitive impairments. Regarding feature extraction methods, Shannon entropy and log energy entropy generally yield superior performance compared to SVD across the classes, suggesting that these methods may be more effective for the analysis of EEG data in this context.

In the following section, we present the classification results obtained through the application of the Naïve Bayes algorithm, as illustrated in Table 3. Consistent with the KNN classification, the rows of this table correspond to the individual proposed quantifiers, which are further subdivided into five sub-rows that represent the respective classes under consideration. The table columns display the mean and standard deviation values for various classification metrics, including accuracy, sensitivity, specificity, F1 score, and precision. Table 3 offers a comprehensive overview of the results derived from the Naïve Bayes classification model, utilizing data from different EEG frequency bands.

The application of the Naïve Bayes classifier to the delta band demonstrated that the highest classification accuracy was attained for Class 2 (MCI) utilizing Shannon entropy, which resulted in an accuracy of 88.56%, a sensitivity of 88.65%, an F1 score of 93.91%, and a precision of 100%. Furthermore, Class 2 was effectively classified using SVD and log energy entropy, achieving accuracies of 88.31% and 88.23%, respectively. These findings suggest that the delta band is advantageous for Class 2 classification. In the context of SVD, the sensitivity, F1 score, and precision were recorded at 88.31%, 93.79%, and 100%, respectively. For log energy entropy, the corresponding metrics were 88.23% for sensitivity, 93.75% for F1 score, and 100% for precision. Following Class 2, the next highest performance was observed for Class 3, with the peak accuracy achieved through Shannon entropy, yielding values of 88.36% for accuracy, 90.34% for sensitivity, 93.68% for F1 score, and 98.37% for precision. Conversely, the lowest recognition rate was noted for Class 4, which exhibited an accuracy of 59.66% when assessed using Shannon entropy.

In the theta band, the highest classification accuracy for Class 2 was achieved through the application of log energy entropy, yielding an accuracy of 88.79%, a sensitivity of 89.92%, an F1 score of 93.94%, and a precision of 98.66%. Additionally, the Naïve Bayes classifier effectively identified Class 2 utilizing SVD and Shannon entropy, resulting in accuracies of 87.53% and 87.52%, respectively. These findings align with those observed in the delta band, indicating that the theta band is particularly effective for Class 2 classification. The next highest performance was recorded for Class 3, where Shannon entropy, log energy entropy, and SVD all yielded identical accuracy values of 88.23%. Conversely, the lowest recognition rate within the theta band was recorded for Class 4, which exhibited an accuracy of 47.16% when assessed using Shannon entropy.

In the alpha band, the highest classification accuracy for Class 2 was attained through the application of log energy entropy, resulting in an accuracy of 88.46%, a sensitivity of 88.49%, an F1 score of 93.86%, and a precision of 100%. Additionally, the Naïve Bayes classifier demonstrated effective recognition of Class 2 when utilizing SVD and Shannon entropy, achieving accuracies of 88.23% and 88.31%, respectively. Class 3 exhibited the next highest performance, with SVD yielding an accuracy of 88.31%. Conversely, the recognition rate was observed for Class 4, which achieved an accuracy of 61.35% when employing Shannon entropy.

The SMR band achieved the highest classification accuracy for Class 2 utilizing Singular Value Decomposition (SVD), which resulted in an accuracy of 89.28%, a sensitivity of 89.23%, an F1 score of 94.26%, and a precision of 100%. Additionally, the Naïve Bayes classifier identified Class 2 using Shannon entropy and log energy entropy, attaining accuracies of 87.25% and 88.55%, respectively. The subsequent best performance was observed for Class 3, where Shannon entropy yielded an accuracy of 88.47%. Conversely, the lowest recognition rate for the SMR band was recorded for Class 4, which exhibited an accuracy of 61.58% when assessed using log energy entropy.

In the beta band, the highest classification accuracy was achieved for Class 3 utilizing Shannon entropy, which resulted in an accuracy of 88.40%, a sensitivity of 91.26%, an F1 score of 93.82%, and a precision of 100%. Additionally, the Naïve Bayes classifier identified Class 3 through SVD and log energy entropy, attaining an accuracy of 88.23%. The subsequent best performance was observed for Class 2, with Shannon entropy yielding an accuracy of 88.38%. Conversely, the lowest recognition rate was recorded for Class 4, with an accuracy of 52.02% when employing Shannon entropy.

In the gamma band, the highest classification accuracy was attained through the application of SVD, resulting in an accuracy of 89.04%, a sensitivity of 88.95%, an F1 score of 94.15%, and a precision of 100%. Additionally, the Naïve Bayes classifier successfully identified Class 3 utilizing log energy entropy and Shannon entropy, achieving accuracies of 88.23% and 88.62%, respectively. Following Class 3, the best performance for Class 2 was recorded using log energy entropy, which yielded an accuracy of 88.23%. Conversely, the lowest recognition rate was noted for Class 5, with an accuracy of 37.85% when employing Shannon entropy.

The results presented in Table 3 indicate that Class 2 (MCI) consistently achieved the highest accuracy across all frequency bands and feature extraction methods. Notably, strong performance was observed when utilizing Shannon and log energy entropy. Class 3 (depression) demonstrated promising results, especially within the beta and gamma bands; however, its performance was less consistent with Class 2. Class 4 (controls) consistently exhibited the lowest accuracy, suggesting difficulties in differentiating healthy individuals from those with cognitive impairments. The analysis of the Naïve Bayes classifier revealed that Shannon entropy and log energy entropy generally outperformed SVD, highlighting their superior effectiveness in analyzing EEG data across various classifications. These findings emphasize the critical role of feature extraction methods in improving classification accuracy for detecting cognitive impairments using EEG data.

In the following section, we present the classification results obtained through the SVM algorithm, as outlined in Table 4. Similar to the KNN classification, the rows of this table correspond to the individual proposed quantifiers, each further subdivided into five sub-rows that represent the respective classes under consideration. The columns display the mean and standard deviation values for various classification metrics, including accuracy, sensitivity, F1 score, and precision. Table 4 offers a comprehensive overview of the results derived from the SVM classification model, utilizing data from different EEG frequency bands.

The analysis of EEG frequency bands yielded several key findings concerning their efficacy in differentiating between MCI and depression. The delta, theta, alpha, SMR, and beta bands demonstrated a maximum accuracy of 88.23% for MCI and depression across various methodologies, including Shannon entropy, log energy entropy, and SVD. This consistent performance across these frequency bands suggests that their features are comparably reliable for distinguishing between the two conditions. In contrast, the gamma band exhibited a marginally higher accuracy of 88.39% for MCI when employing Shannon entropy, indicating a potential advantage in identifying MCI relative to depression.

Table 5 delineates the classification outcomes achieved through the application of the AdaBoost algorithm. Consistent with the prior analyses, the rows of this table represent the various proposed features, each further segmented into five sub-rows that correspond to the respective classification groups. The columns display the mean and standard deviation values for several regression metrics, including accuracy, sensitivity, F1 score, and precision. This table summarizes the results from the AdaBoost model, utilizing different EEG frequency band data.

The analysis of EEG features across various frequency bands demonstrated that in the delta band, an accuracy of 88.23% was achieved utilizing Shannon entropy, log energy entropy, and SVD features for Class 2 and Class 3. The theta band also yielded an accuracy of 88.23% with the same features for the same classes. In the alpha band, log energy entropy attained the highest accuracy of 88.31%, specifically for Class 3. The SMR band achieved the highest accuracy of 88.40% with log energy entropy for Class 2. In the beta band, Shannon entropy, log energy entropy, and SVD features all recorded an accuracy of 88.23% for Class 2 and Class 3. Finally, in the gamma band, Shannon entropy and SVD features produced the highest accuracy of 88.97% for Class 3. These findings underscore the effectiveness of the AdaBoost algorithm in accurately classifying cognitive states, particularly MCI, depression, and Alzheimer’s disease, based on EEG features across different frequency bands.

In the subsequent section, we introduce an alternative methodology for classification. Whereas the preceding section concentrated on the independent categorization and classification of individual features, this section integrates all features into a unified classification module. For this analysis, we employed the KNN algorithm, as delineated in Table 6. Consistent with the prior section, the rows in the table correspond to five distinct classes, each further divided into five sub-rows. The columns present the average and standard deviation values for various classification metrics, including accuracy, sensitivity, specificity, and precision.

Table 6 provides a detailed summary of the results obtained from the KNN classification model, which incorporates data from various EEG bands and fused features.

The highest accuracy rate of 88.38% for Class 2 is attained through the utilization of delta band measures, with corresponding values for sensitivity, F1 score, and precision recorded at 88.87%, 93.80%, and 99.80%, respectively.

For Class 2, an accuracy of 88.75% is achieved utilizing theta band measures, accompanied by sensitivity, F1 score, and precision values of 89.32%, 93.96%, and 99.52 ± 0.50%, respectively. In contrast, an accuracy rate of 88.90% for Class 2 is achieved using alpha band measures, with corresponding sensitivity, F1 score, and precision values of 89.32%, 94.06%, and 100%, respectively. Likewise, when employing SMR band measures, Class 2 attains a maximum accuracy rate of 88.61%, with sensitivity, F1 score, and precision values of 88.84%, 93.93%, and 100%, respectively.

For Class 2, the highest accuracy rate of 88.85% is achieved through the utilization of beta band measures, corresponding to sensitivity, F1 score, and precision values of 89.21%, 94.04%, and 99.90%, respectively. Conversely, Class 3 attains the highest accuracy rate of 89.04% by employing gamma band measures, with sensitivity, F1 score, and precision values of 89.98%, 94.13%, and 99.71%, respectively.

Table 6 indicates that Classes 2 and 3 display superior classification performance across all EEG bands, particularly concerning accuracy, sensitivity, and precision. In contrast, Class 4 demonstrates the lowest accuracy, suggesting significant challenges in classification. Meanwhile, Class 5 yields moderate results; however, it does not achieve the performance levels of Classes 2 and 3.

The following section presents the classification results obtained using the Naïve Bayes algorithm, as summarized in Table 7. Consistent with the previous analyses, the rows are divided into five sub-rows, each representing one of the classified classes. The columns illustrate the average and standard deviation values for the classification metrics, which include accuracy, sensitivity, specificity, and precision. Specifically, Table 7 delineates the results of Naïve Bayes, employing various EEG band data and fused features.

For Class 2, the highest accuracy of 87.18% is achieved using delta band measures, with corresponding sensitivity, F1 score, and precision values of 89.94%, 93.05%, and 97.42%, respectively. When employing theta band measures for Class 2, the accuracy increases to 88.05%, accompanied by sensitivity, F1 score, and precision values of 90.91%, 93.44%, and 97.22%, respectively. For Class 2, the highest accuracy achieved using alpha band measures is 86.43%, with corresponding sensitivity, F1 score, and precision values of 92.11%, 92.56%, and 96.46%, respectively.

In the analysis of Class 3, the highest accuracy achieved is 87.93% when employing SMR band measures, accompanied by sensitivity, F1 score, and precision values of 90.02%, 93.55%, and 99.32%, respectively. For Class 2, the utilization of beta band measures for Class 2 results in an accuracy rate of 84.24%, with corresponding sensitivity, F1 score, and precision values of 91.86%, 91.15%, and 92.74%. Finally, the implementation of gamma band measures for Class 3 produces an accuracy of 88%, with sensitivity, F1 score, and precision values of 89.34%, 93.56%, and 98.94%, respectively.

The findings illustrated in Table 7 indicate that Class 2 consistently surpasses the performance of the other classes, especially within the delta and theta frequency bands. Conversely, Class 3 demonstrates robust performance in the SMR and gamma bands. In contrast, Classes 1 and 4 exhibit lower accuracy across the majority of frequency bands, suggesting a greater challenge in classification. Notably, the gamma band presents particular difficulties for Classes 2 and 5.

Table 8 presents the outcomes of the SVM classification utilizing various EEG frequency band data alongside combined features.

The classification outcomes for the SVM classifier that employed EEG frequency band data indicate high accuracy across various classes and frequency bands. Classes 2 and 3 consistently achieved the highest accuracy rate of 88.23% across the delta, theta, alpha, SMR, and beta bands, with corresponding F1 scores of 93.75% and precision values of 100%. In the gamma band, Class 3 exhibited a significant improvement, attaining an accuracy of 89.06%, a sensitivity of 88.97%, an F1 score of 94.16%, and a precision of 100%. Similarly, Class 5 in the gamma band also exhibited a high accuracy of 83.13%, maintaining its strong performance across all frequency bands. These findings highlight the robustness of the SVM classifier in effectively distinguishing critical EEG features, particularly in Classes 2, 3, and 5.

Table 9 illustrates the classification results obtained using the AdaBoost algorithm.

The delta band exhibits the highest accuracy of 88.23% in distinguishing between Class 2 and Class 3. The theta band attained the best accuracy of 88.63% in Class 2. Similarly, the alpha band demonstrates the highest accuracy of 88.23% for Classes 2 and 3. In the SMR band, Class 2 attained a peak accuracy of 88.63%. The beta band records a maximum accuracy of 88.31% for Class 2. Notably, the gamma band achieves the highest accuracy of 89.05% in Class 3.

Among the various frequency bands, the gamma band demonstrates the highest overall performance, achieving an accuracy of 89.05% in Class 3. This finding positions the gamma band as the most accurate frequency band evaluated. Such results underscore the potential of the gamma band for enhancing classification performance in EEG-based applications utilizing the AdaBoost algorithm.

**Figure 4:**
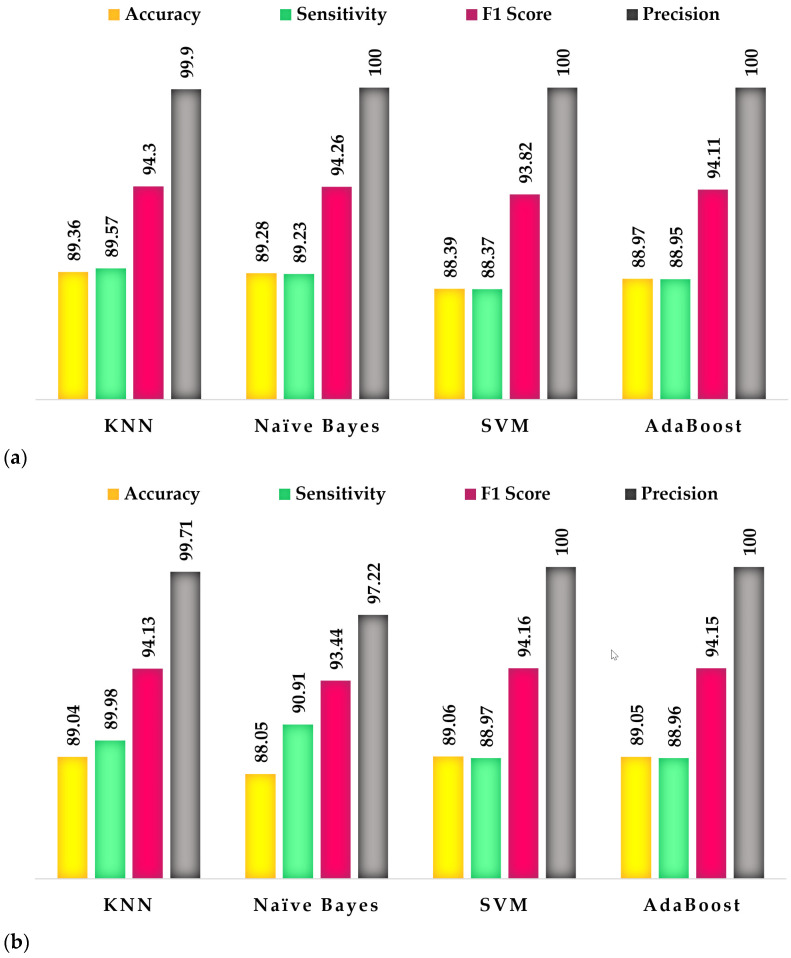
The optimal classification rates: (**a**) without combined features and (**b**) with combined features.

Figure 4 presents the optimal classification results obtained by each classifier, as outlined in Table 6, Table 7, Table 8 and Table 9, which include feature combinations, and Table 2, Table 3, Table 4 and Table 5, which do not incorporate feature combinations.

All computations were conducted on a system with the following specifications: an 11th Generation Intel(R) Core (TM) i7-11700 processor operating at 2.50 GHz. The computational time required for various classification algorithms exhibited variability contingent upon the method employed, the frequency bands analyzed, and the specific features utilized. Notably, the processing time for the SVM classifier was 19.21 s per frequency band for each feature. In contrast, the AdaBoost classifier required a longer processing duration of 69.17 s per frequency band. The Naïve Bayes classifier demonstrated a relatively shorter computation time of 18.11 s, while the KNN classifier necessitated 26.84 s per frequency band for each feature. Additionally, the computational cost associated with the extraction of the Granger matrix and the LGS features across all frequency bands for each participant was 56.34 s.

## 4. Discussion

This study examines the classification of cognitive and psychological disorders using algorithms that utilize brain connectivity, with a specific emphasis on Granger causality and local graph structures. The proposed methodology encompasses several critical steps, including the preprocessing of EEG signals, extraction of frequency bands, and analysis of Granger causality. Following these initial steps, feature extraction is conducted via local structure graphs to quantify the data. Three indices were utilized for feature extraction: logarithmic energy entropy, Shannon entropy, and the largest singular value. The extracted features were subsequently classified using various classifiers, including KNN, SVM, AdaBoost, and Naïve Bayes. The efficacy of the proposed method was evaluated using performance metrics such as accuracy, sensitivity, F1 score, and precision. Our findings, as detailed in Table 2, reveal that the KNN classifier, employing SVD for Class 3 (depression), achieved the highest accuracy in the gamma band, with an accuracy of 89.36%, a sensitivity of 89.57%, an F1 score of 94.30%, and a precision of 99.90%. The second-best results, presented in Table 3, were obtained using the Naïve Bayes classifier, which achieved the highest accuracy in the SMR band for Class 2 (MCI) with SVD, resulting in an accuracy of 89.28%, a sensitivity of 89.23%, an F1 score of 94.26%, and a precision of 100%. Overall, the results indicate that the KNN classifier outperformed all other classifiers in this study.

Furthermore, when all features were integrated, our analysis, as presented in Table 8, concerning the SVM classifier, reveals that the highest accuracy in the gamma band was observed for Class 3 (depression), achieving an accuracy of 89.06%, a sensitivity of 88.97%, an F1 score of 94.16%, and a precision of 100%. The second-best performance was recorded by the AdaBoost classifier, as detailed in Table 9, which also demonstrated the highest accuracy in the gamma band for Class 3 (depression), with an accuracy of 89.05%, a sensitivity of 88.96%, an F1 score of 94.15%, and a precision of 100%.

Our findings reveal significant differences in EEG rhythms across the cognitive and psychological disorders investigated. In the case of MCI, the Naïve Bayes classifier demonstrated an accuracy of 89.28% within the SMR band. Previous research indicates that SMR is associated with sensory processing and attentional mechanisms [45,46], which are frequently disrupted in patients with MCI. Furthermore, studies have shown that SMR neurofeedback training can enhance cognitive performance in elderly individuals diagnosed with MCI [47,48]. The preservation of SMR activity in MCI patients may reflect a compensatory mechanism, enabling them to sustain certain sensory processing capabilities despite cognitive decline. This observation aligns with theoretical frameworks suggesting that individuals experiencing early cognitive impairments may increasingly depend on intact sensory processing networks. For instance, the prior literature has documented disruptions in the functional brain networks of MCI patients [49], particularly emphasizing deficits in functional connectivity within the default mode network. In this context, sensory processing networks may serve a compensatory function, helping to alleviate the effects of diminished connectivity in MCI. The highest accuracy for the KNN, SVM, and AdaBoost classifiers was recorded in the gamma band for depression. This finding is consistent with the existing literature that identifies gamma rhythms as potential biomarkers or endophenotypes for major depression [50]. Additionally, it corroborates studies that associate heightened gamma activity with cognitive processing and working memory tasks [51]; meanwhile, diminished gamma activity has been observed in depressed patients, indicating impaired cognitive function and emotional processing [52]. As previously noted, gamma rhythms are linked to higher-order cognitive functions, including attention and working memory. The significant findings in the gamma band for depression suggest a relationship between altered cognitive processing and the emotional disturbance characteristic of this disorder. Moreover, the highest accuracy (88.05%) for the Naïve Bayes classifier in the theta band was also noted for MCI. The literature indicates that increased theta activity is generally associated with cognitive impairment, particularly in relation to attention, working memory, and processing speed, as observed in participants with Alzheimer’s disease [53,54,55] and those exhibiting decreased cognitive functioning [56]. Andreou et al. [57] posited that enhanced theta-band connectivity during resting states may be linked to the over-activation of the default mode network, potentially leading to memory impairments due to the insufficient modulation of theta-band activity during memory retrieval episodes. Our results support this hypothesis, as MCI is characterized by deficits in these cognitive domains, resulting in altered theta dynamics. The elevation of theta activity has been documented in conditions characterized by cognitive impairment, as this often signifies increased cognitive load and challenges sustaining attention. This is particularly pertinent in MCI, where attentional resources are frequently compromised.

Previous research has predominantly concentrated on the diagnosis of cognitive and mental disorders (refer to Table 10). In contrast, the algorithm proposed in this study is designed for multi-class classification, while most of the studies reviewed are limited to binary (two-class) classification. A comparison of these studies is provided in Table 10.

The research conducted by Ciprian et al. [16] involved a sample of 62 individuals diagnosed with schizophrenia and 70 healthy control participants. The study employed feature extraction techniques, specifically the computation of symbolic transfer entropy and the Relief algorithm, to identify distinguishing features. Various classifiers were utilized, including Gaussian Naïve Bayes, linear discriminant analysis, KNN, Support Vector Machine (SVM), and random forest (RF). The findings revealed that the KNN algorithm demonstrated the highest performance, achieving an accuracy of 92.96%. This finding underscores the efficacy of the KNN method in the diagnosis of psychological disorders.

Godfrey and Singh [15] conducted an examination involving 34 individuals diagnosed with depression and 30 healthy control participants. They utilized a range of feature extraction techniques, which included statistical analysis, spectral analysis, wavelet analysis, and functional connectivity assessments. Additionally, feature selection was performed, and classifiers such as Linear Support Vector Machine (LINSVM), Radial Basis Function Support Vector Machine (RBFSVM), and RF were implemented. The findings indicated that RBFSVM yielded the highest performance, achieving an accuracy rate of 99% and demonstrating the most significant differences within the delta band.

In the study conducted by Ruiz de Miras et al. [17], 11 individuals diagnosed with schizophrenia and 20 healthy control participants were examined. The researchers identified 17 linear and nonlinear criteria for feature extraction. A range of classification algorithms, including KNN, logistic regression, decision trees, random forest (RF), and SVM, were employed to classify the data. The results indicated that the KNN algorithm demonstrated the highest performance, achieving an accuracy rate of 87%.

The research conducted by Shen et al. [18] involved a sample of 45 individuals diagnosed with schizophrenia and 39 healthy control participants. In this study, a multi-variate autoregressive model was utilized to transform the data into frequency-domain features within the alpha band. Various algorithms, including KNN, SVM, decision trees, and three-dimensional convolutional neural networks (3D-CNN), were implemented. The findings revealed that the 3D-CNN model demonstrated superior performance, achieving an accuracy rate of 98.47%.

In a separate study [58], 15 individuals diagnosed with schizophrenia and 14 healthy control participants were examined. This investigation assessed various criteria, including degree D, clustering coefficient, global efficiency, local efficiency, and betweenness centrality, by calculating the weighted phase delay index through fuzzy correlation and feature analysis. The findings revealed a statistically significant difference in AUC values within the temporal lobe regions.

The research conducted by Shan et al. [19] examined a cohort comprising 19 individuals diagnosed with Alzheimer’s disease and 20 healthy control participants. The study employed six functional connectivity measures, namely Pearson correlation, wavelet correlation, squared large correlation, phase synchronization, phase locking, and phase delay index, in conjunction with spatial–temporal graph convolutional neural networks and temporal convolutional neural networks. The findings revealed that the accuracy of the spatial–temporal graph convolutional neural network was significantly higher in the “closed-eye” condition compared to the “open-eye” condition. A separate investigation [20] analyzed a cohort comprising 13 individuals diagnosed with mild cognitive impairment and 20 healthy controls. The researchers computed the weighted connectivity index across various frequency bands and employed the minimum spanning tree algorithm to extract features, including degree, leaf composition, diameter, oval distance, betweenness centrality, and tree hierarchy. The findings revealed a statistically significant reduction in average power within the alpha and beta frequency bands, indicating a low-integrated system characterized by diminished data transmission efficiency identified in the patient group.

A separate study [24] investigated a cohort comprising 20 individuals diagnosed with Alzheimer’s disease and 20 healthy controls. The researchers extracted various features, including Pearson correlation, spectral correlation, section correlation, and weighted delay indices. The algorithms utilized in this research included GCN, SVM, and CNN. The findings revealed that GCN demonstrated a superior AUC to the other algorithms.

In the study conducted by Rodriguez-Gonzalez et al. [59], data were collected from 44 individuals diagnosed with mild cognitive impairment, 50 individuals with Alzheimer’s disease, and 67 control participants. The researchers employed MEG to extract meta-band features, focusing on critical characteristics such as absorption power, dominance degree, topological matching, displacement rate, and band complexity. This investigation resulted in three primary meta-bands and highlighted significant differences between the healthy individuals and those in the patient groups.

Two advanced methodologies utilized the same publicly accessible EEG dataset [33]. Shor et al. [60] and Benninger et al. [33] proposed ultra-metric analyses associated with p-adic numbers and quantum theory to quantify brain connectivity in both spatial and temporal dimensions, as well as to assess the instantaneous non-local effects within information space. The quantum potential means and variability score (QPMVS) approach exhibited robust performance, depicted as the area under the curve (AUC) of the receiver operating characteristic (ROC) curves. The authors reported an AUC of 0.8992 for distinguishing schizophrenia from depression. When differentiating schizophrenia from Alzheimer’s disease, the AUC was 0.8762, and for the comparison of schizophrenia versus mild cognitive impairment (MCI), the AUC was 0.8914. Similarly, the method achieved an AUC of 0.8777 for differentiating depression from Alzheimer’s disease, and an AUC of 0.8929 for depression versus MCI. Furthermore, comparisons between Alzheimer’s disease and MCI yielded an AUC of 0.7660. The QPMVS method also effectively distinguished neuropsychiatric patient groups from healthy controls, with an AUC of 0.8981 for control versus schizophrenia, an AUC of 0.9033 for control versus depression, an AUC of 0.9143 for control versus Alzheimer’s disease, and an AUC of 0.8309 for control versus MCI. Shor et al. [60] employed an innovative mathematical methodology grounded in p-adic number theory to differentiate between patients diagnosed with schizophrenia and those with major depression in comparison to healthy controls. This study utilized electroencephalogram (EEG) signals from a total of 166 participants (28 with major depression, 42 with schizophrenia, and 95 controls). The authors focused on the spatiotemporal relationships of individual EEG electrode signals, characterizing these relationships through distinct topological structures, specifically the personal universal dendrographic hologram and the personal block dendrographic hologram signature. The findings revealed that the relational topological structures exhibited unique patterns corresponding to each diagnostic group, achieving remarkable classification performance with an area under the curve (AUC) of 0.9908 for the comparison between controls and schizophrenia, and 0.9986 for the comparison between controls and depression. It is noteworthy that this study utilized a dataset similar to ours, albeit limiting the classification to three groups rather than five, thereby underscoring the efficacy of their methodology in providing an objective diagnostic tool for psychiatric disorders.

In comparing the results of our proposed method with those of prior studies focused on the detection of cognitive and mental disorders (Table 10), several key advantages and strengths of our approach are evident. Many previous studies are limited to binary classification (e.g., differentiating between patients and controls), whereas our method addresses a more complex multi-class classification problem. Specifically, we distinguish among five distinct classes: major depression, schizophrenia, Alzheimer’s disease, mild cognitive impairment, and healthy controls. This makes our approach more versatile and applicable to a broader range of clinical applications compared to the binary models presented in studies such as [15,16,17].

Our method achieves a classification accuracy of 89.36%, which compares favorably to several existing approaches. For instance, the KNN method reported in [16] achieved an accuracy of 92.96%, but the study was restricted to a binary classification of schizophrenia versus healthy controls. In [17], the best KNN accuracy was 87%, again involving a binary classification with schizophrenia patients. Notably, our results demonstrate higher accuracy than some studies that concentrate on specific cognitive disorders, such as the 92.3% accuracy reported for Alzheimer’s patients in [19], utilizing spatio-temporal graph convolutional networks (ST-GCNs).

Unlike many previous studies that centered on specific feature extraction methods or limited feature sets (e.g., statistical or spectral analysis in [15], or phase lag index in [58]), we employed a broader range of features from various EEG frequency bands. Additionally, we integrated Granger causality and local graph structures, which provide a more comprehensive understanding of the brain’s functional connectivity and enhance classification performance. Our study utilized a considerably larger dataset than many reviewed studies, comprising 230 participants. This diverse sample included a significant number of subjects with different mental health conditions, enhancing the robustness and generalizability of our approach. In contrast, several studies, such as [58] (15 patients) and [59] (44 patients with mild cognitive impairment), used smaller sample sizes, potentially compromising the reliability of their results.

We employed several widely recognized and effective classification algorithms, including KNN, SVM, AdaBoost, and Naïve Bayes. While certain studies, such as those referenced in [18,19], have utilized more complex methodologies, such as 3D-CNN and ST-GCN, our approach achieves competitive accuracy with simpler models. This characteristic enhances accessibility in clinical environments where computational resources may be constrained. Furthermore, our method integrates multiple EEG frequency bands, which have been demonstrated to capture a broader spectrum of brain activity. In contrast, studies such as [59] have concentrated on a limited number of frequency bands (e.g., three primary meta-bands), whereas our approach leverages a more extensive range of EEG data, thereby providing a richer dataset for the classification task.

While the present study has yielded promising results, it is essential to acknowledge certain limitations. First, although our dataset comprises 230 participants, this sample size may still be considered insufficient for certain mental health conditions, such as major depression (28 individuals) and schizophrenia (42 individuals). A larger and more diverse dataset would enhance the generalizability of the findings, particularly across various populations, age groups, and cultural contexts. Furthermore, to address the issue of limited data for specific mental health conditions, such as schizophrenia and major depression, future studies should consider employing data augmentation strategies. Second, the study utilized the one-versus-all (OVA) classification technique, commonly employed to resolve multiclass classification problems by converting them into multiple binary classification tasks. However, it is important to recognize that the OVA approach may present challenges due to imbalanced class distribution, particularly when certain classes contain significantly more samples than others. Additionally, classification errors in one class can negatively impact the performance of other classifications. Therefore, future studies are encouraged to explore alternative classification methods, such as multiclass and one-versus-one approaches, to provide a more accurate evaluation of the proposed model’s validity. Third, the method incorporates various features from different EEG frequency bands, which increases the computational complexity of the model. While this approach captures a broader range of brain activity, it also necessitates greater processing power, potentially limiting its efficiency for real-time applications. To mitigate this computational complexity, future research should focus on feature selection or dimensionality reduction techniques. Moreover, future research could involve closer collaboration with neurologists, psychologists, and other domain experts to enhance our understanding of which features of EEG data are most relevant for specific mental health conditions. This collaboration could lead to more targeted and efficient feature extraction processes that focus on the most significant biomarkers of cognitive and mental disorders, thereby optimizing the model’s performance and reducing unnecessary complexity. (4) A significant limitation of the present study is the absence of comprehensive information regarding the pharmacological therapies administered to participants before EEG recordings. Specifically, the dataset utilized [33] did not include data on whether patients were receiving any medications before the EEG sessions, nor did it specify the duration and types of medications that may have been administered. This limitation may substantially impact the conclusions drawn from our study, as pharmacological interventions can exert considerable effects on brain activity and EEG patterns. In the absence of knowledge regarding the medications and their potential influence on the EEG results, it becomes challenging to determine whether the observed differences in brain activity across the groups are attributable solely to the neuropsychiatric conditions under investigation or are confounded by prior medication effects. Future research should prioritize the collection and integration of comprehensive medication histories, including detailed information on the types of medications administered, their dosages, and the duration of treatment before EEG sessions. Such studies would facilitate the delineation of the effects of specific medications on EEG outcomes, thereby enhancing the interpretability of the results. (5) Another notable limitation of our study is the lack of specific artifact rejection procedures during the preprocessing of EEG data. While our approach aimed to streamline the data processing workflow and reduce complexity, this decision may have compromised our ability to enhance classification accuracies. In the absence of thorough artifact rejection, residual noise from muscle activity and eye blinks could confound the neural signals of interest, thereby affecting the robustness and reliability of our findings. The inclusion of preprocessing steps specifically targeting artifact removal could improve the quality of the EEG data and lead to more accurate classification outcomes. In light of this limitation, we recommend that future research explore the implementation of advanced artifact removal techniques to effectively isolate and eliminate artifacts. (6) The number of channels in an EEG setup can significantly influence various aspects of the proposed methodology, including data acquisition, signal quality, and the performance of classification models. The number of channels directly affects the dimensionality of the data; an increased number of channels can provide a more comprehensive representation of brain connectivity and dynamics, potentially leading to improved feature extraction and classification accuracy. However, it is also important to acknowledge that an increase in the number of channels may introduce challenges such as heightened noise and increased computational complexity. In our study, we employed a 19-channel EEG system. Future research should include comparisons with various EEG setups and configurations, utilizing a greater number of channels (e.g., 32 or 64 channels) to assess how these changes influence the robustness and accuracy of our classification algorithms.

## 5. Conclusions

This study introduces a novel framework for the diagnosis of multiple mental disorders through analyzing brain connectivity. Our methodology involves the extraction of EEG frequency bands, which allows for the decomposition of signals into five distinct brain rhythms. Subsequently, Granger causality is calculated from these rhythms, leading to the extraction of various local graph structures and nonlinear indices. A range of machine learning algorithms are then applied to identify five mental disorders, including schizophrenia, mild cognitive impairment (MCI), Alzheimer’s disease, and depression, as well as to distinguish healthy controls. The experimental results indicate that the KNN classifier exhibits superior performance, particularly in detecting depression within the gamma band, achieving an accuracy of 89.36%. This finding highlights the advantages of our multi-class classification approach compared to the traditional binary classifications prevalent in the existing literature. With commendable accuracy, sensitivity, and precision metrics, our results advocate for the integration of these computational techniques into clinical practice, facilitating earlier and more accurate diagnoses to mitigate the adverse effects associated with delayed diagnosis and treatment.

The methodology we propose exhibits significant potential; however, it is not devoid of limitations. These limitations encompass its dependence on a specific dataset, the characteristics associated with EEG setups and configurations, as well as the complexities inherent in the multi-stage processing approach. It is imperative to address these challenges to facilitate the wider adoption of our strategy in clinical practice. Future research should prioritize the validation of our methodology using independent datasets to evaluate its generalizability and robustness. Furthermore, the integration of advanced deep learning models with our existing signal processing techniques may yield valuable insights and enhance the identification of more optimal methodologies.

## Figures and Tables

**Table 1:** Demographics of participants.

Group			Number	Female/Male	Age (Mean ± SD)	Age Range
Schizophrenia			42	15/27	41.4 ± 16.8	18–76
Depression			28	20/8	69.7 ± 14.8	33–91
Control			95	63/32	52.2 ± 16.8	19–80
Cognitive Decline			65	31/34	72.9 ± 7.2	60–87
	Alzheimer’s Disease		40	20/20	72.7 ± 7.9	60–87
	Mild Cognitive Impairment (MCI)		25	11/14	73.5 ± 6.0	62–85
		Stable MCI	6	0/6	74.3 ± 4.6	67–80
		Deteriorating MCI	9	6/3	73.2 ± 5.6	65–82

**Table 2:** KNN classification rates utilizing different EEG frequency band features.

Feature	Class Number	Accuracy (%)	Sensitivity (%)	F1 Score (%)	Precision (%)
EEG δ band data					
Shannon entropy	1	81.77 ± 0.70	83.57 ± 0.24	89.83 ± 0.4	98.36 ± 0.71
	2	88.55 ± 0.45	89.01 ± 0.29	93.90 ± 0.22	100 ± 0.0
	3	88.55 ± 0.43	88.94 ± 0.32	93.89 ± 0.20	99.81 ± 0.0
	4	60.22 ± 1.49	64.96 ± 1.12	68.97 ± 1.53	74.96 ± 2.1
	5	81.39 ± 0.56	82.71 ± 0.41	89.66 ± 0.33	98.45 ± 0.72
Log energy entropy	1	82.49 ± 0.53	84.03 ± 0.52	90.23 ± 0.27	98.36 ± 0.64
	2	88.46 ± 0.54	89.02 ± 0.30	93.84 ± 0.27	99.71 ± 0.40
	3	88.63 ± 0.43	89.06 ± 0.43	93.93 ± 0.43	100 ± 0.0
	4	60.46 ± 1.64	64.94 ± 1.87	68.99 ± 1.41	75.10 ± 2.38
	5	81.56 ± 0.85	83.22 ± 0.64	89.69 ± 0.41	98.45 ± 0.69
SVD	1	81.73 ± 1.00	82.95 ± 0.38	89.88 ± 0.57	98.47 ± 0.69
	2	87.97 ± 0.25	88.46 ± 0.45	93.60 ± 0.13	99.71 ± 0.30
	3	88.61 ± 0.40	88.98 ± 0.23	93.93 ± 0.21	99.90 ± 0.49
	4	59.20 ± 2.13	63.60 ± 1.49	68.62 ± 1.83	75.86 ± 3.05
	5	81.01 ± 0.67	82.83 ± 0.53	89.39 ± 0.40	97.94 ± 0.97
EEG θ band data					
Shannon entropy	1	82.87 ± 1.00	83.93 ± 0.54	90.46 ± 0.51	99.08 ± 0.64
	2	88.45 ± 0.43	88.82 ± 0.48	93.84 ± 0.20	99.81 ± 0.30
	3	88.64 ± 0.50	89.04 ± 0.32	93.94 ± 0.26	99.90 ± 0.10
	4	61.41 ± 1.00	65.23 ± 1.23	70.69 ± 0.97	79.18 ± 2.51
	5	82.19 ± 0.78	83.29 ± 0.63	90.06 ± 0.48	98.56 ± 0.99
Log energy entropy	1	81.86 ± 0.72	83.38 ± 0.27	89.90 ± 0.44	98.26 ± 0.96
	2	88.36 ± 0.35	89.03 ± 0.34	93.79 ± 0.18	99.71 ± 0.29
	3	89.29 ± 0.68	89.74 ± 0.74	94.27 ± 0.33	100 ± 0.0
	4	60.12 ± 2.03	64.20 ± 1.56	69.24 ± 1.75	76.50 ± 2.46
	5	81.57 ± 1.38	83.22 ± 0.78	89.67 ± 0.75	98.04 ± 0.86
SVD	1	83.16 ± 0.84	83.94 ± 0.64	90.66 ± 0.43	99.39 ± 0.52
	2	88.36 ± 0.59	89.19 ± 0.39	93.81 ± 0.32	99.90 ± 0.10
	3	88.80 ± 0.45	89.05 ± 0.35	94.01 ± 0.23	99.90 ± 0.10
	4	63.26 ± 0.99	66.48 ± 1.04	71.82 ± 1.02	80.04 ± 2.19
	5	80.90 ± 0.98	83.28 ± 0.52	89.280.61	97.42 ± 1.09
EEG α band data					
Shannon entropy	1	82.22 ± 0.62	83.46 ± 0.53	90.16 ± 0.32	99.08 ± 0.53
	2	88.63 ± 0.58	89.03 ± 0.41	93.92 ± 0.29	99.81 ± 0.20
	3	88.79 ± 0.47	89.25 ± 0.47	94.01 ± 0.24	99.90 ± 0.10
	4	60.17 ± 1.17	64.83 ± 1.03	68.73 ± 1.27	75.11 ± 2.37
	5	81.29 ± 0.68	82.79 ± 0.47	89.59 ± 0.40	98.14 ± 0.76
Log energy entropy	1	83.33 ± 0.96	85.23 ± 0.51	90.62 ± 0.53	98.16 ± 0.98
	2	89.26 ± 0.47	89.47 ± 0.44	94.25 ± 0.24	100 ± 0.0
	3	88.37 ± 0.25	88.68 ± 0.34	93.81 ± 0.14	99.90 ± 0.10
	4	58.69 ± 2.66	63.49 ± 1.18	68.17 ± 1.79	75.10 ± 1.34
	5	82.66 ± 1.1	83.82 ± 0.77	90.28 ± 0.57	98.97 ± 0.43
SVD	1	82.69 ± 0.72	83.61 ± 0.31	90.39 ± 0.42	99.18 ± 0.64
	2	88.88 ± 0.53	89.25 ± 0.43	94.06 ± 0.26	99.81 ± 0.0
	3	88.64 ± 0.59	89.17 ± 45	93.93 ± 0.30	99.90 ± 0.30
	4	60.99 ± 1.43	65.47 ± 1.26	69.28 ± 1.2	75.13 ± 2.34
	5	82.11 ± 0.73	83.03 ± 0.51	90.05 ± 0.41	98.97 ± 0.65
EEG SMR band data					
Shannon entropy	1	81.60 ± 0.78	82.83 ± 0.36	89.83 ± 0.44	98.87 ± 0.58
	2	88.38 ± 0.34	88.58 ± 0.33	93.82 ± 0.17	100 ± 0.0
	3	89.05 ± 0.39	89.22 ± 0.52	94.15 ± 0.18	100 ± 0.0
	4	57.59 ± 1.51	62.21 ± 0.89	67.61 ± 1.97	76.23 ± 3.11
	5	82.57 ± 0.93	83.35 ± 0.69	90.30 ± 0.49	99.38 ± 0.72
Log energy entropy	1	81.05 ± 0.71	82.86 ± 0.37	89.46 ± 0.40	97.95 ± 0.86
	2	88.39 ± 0.41	88.78 ± 0.22	93.82 ± 0.20	100 ± 0.0
	3	88.94 ± 0.40	89.20 ± 0.35	94.08 ± 0.19	99.90 ± 0.0
	4	60.42 ± 2.56	64.49 ± 2.28	69.81 ± 2.20	78.01 ± 3.09
	5	82.53 ± 0.88	83.38 ± 0.54	90.29 ± 0.57	99.18 ± 0.69
SVD	1	82.07 ± 0.33	83.51 ± 0.53	90.07 ± 0.19	98.88 ± 0.52
	2	88.48 ± 0.45	88.82 ± 0.11	93.86 ± 0.23	100 ± 0.0
	3	88.36 ± 0.56	88.97 ± 0.26	93.79 ± 0.29	99.71 ± 0.39
	4	57.35 ± 2.41	62.33 ± 1.69	66.97 ± 1.84	73.93 ± 2.98
	5	81.81 ± 0.80	83.23 ± 0.39	89.85 ± 0.49	98.45 ± 1.11
EEG β band data					
Shannon entropy	1	81.80 ± 1.05	83.84 ± 0.50	89.82 ± 0.59	98.05 ± 0.68
	2	88.61 ± 0.39	89.11 ± 0.21	93.93 ± 0.19	100 ± 0.0
	3	88.65 ± 0.34	88.81 ± 0.23	93.95 ± 0.17	100 ± 0.0
	4	63.66 ± 1.48	67.97 ± 1.49	71.42 ± 0.90	77.95 ± 2.27
	5	82.23 ± 0.76	83.19 ± 0.41	90.13 ± 0.42	99.28 ± 0.43
Log energy entropy	1	81.60 ± 0.61	83.26 ± 0.52	89.73 ± 0.37	97.95 ± 0.75
	2	89.28 ± 0.85	89.55 ± 0.58	94.26 ± 0.43	99.90 ± 0.0
	3	89.10 ± 0.55	89.14 ± 0.27	94.18 ± 0.28	99.90 ± 0.10
	4	60.84 ± 1.94	66.36 ± 1.76	68.42 ± 1.15	73.52 ± 2.76
	5	82.59 ± 0.81	83.36 ± 0.50	90.31 ± 0.47	99.28 ± 0.54
SVD	1	82.48 ± 0.93	84.24 ± 0.40	90.15 ± 0.56	98.06 ± 1.27
	2	88.61 ± 0.55	89.28 ± 0.50	93.89 ± 0.29	99.52 ± 0.30
	3	88.80 ± 0.73	89.39 ± 0.49	94.01 ± 0.37	99.81 ± 0.0
	4	60.22 ± 1.81	65.03 ± 1.82	68.80 ± 1.06	75.42 ± 1.98
	5	82.37 ± 0.69	82.90 ± 0.29	90.20 ± 0.37	99.38 ± 0.65
EEG γ band data					
Shannon entropy	1	83.88 ± 0.73	84.76 ± 0.58	90.95 ± 0.42	98.97 ± 0.68
	2	88.54 ± 0.70	89.03 ± 0.33	93.88 ± 0.36	99.90 ± 0.10
	3	88.37 ± 0.51	89.20 ± 0.37	93.78 ± 0.26	99.52 ± 0.48
	4	64.30 ± 2.62	67.08 ± 2.14	72.73 ± 1.8	80.76 ± 1.93
	5	81.51 ± 0.93	84.21 ± 0.79	89.51 ± 0.47	96.80 ± 1.02
Log energy entropy	1	83.13 ± 0.81	84.05 ± 0.52	90.59 ± 0.47	99.18 ± 0.64
	2	88.54 ± 0.46	88.77 ± 0.25	93.90 ± 0.23	100 ± 0.0
	3	88.46 ± 0.54	89.04 ± 0.28	93.83 ± 0.30	99.52 ± 0.40
	4	63.60 ± 2.41	67.81 ± 1.81	71.22 ± 1.94	76.31 ± 3.1
	5	82.51 ± 1.07	84.01 ± 0.78	90.15 ± 0.61	98.14 ± 0.94
SVD	1	83.19 ± 1.14	84.67 ± 0.88	90.52 ± 0.58	98.25 ± 0.97
	2	88.87 ± 0.75	89.49 ± 0.43	94.02 ± 0.41	99.52 ± 0.37
	3	89.36 ± 0.57	89.57 ± 0.19	94.30 ± 0.30	99.90 ± 0.10
	4	66.63 ± 2.22	70.06 ± 1.19	74.09 ± 1.67	81.72 ± 3.16
	5	82.30 ± 0.51	84.32 ± 0.76	90 ± 0.30	97.73 ± 1.44

Note—Class 1: schizophrenia; Class 2: mild cognitive impairment (MCI); Class 3: depression; Class 4: controls (healthy individuals); and Class 5: Alzheimer’s disease.

**Table 3:** Naïve Bayes classification rates utilizing different EEG frequency band features.

Feature	Class Number	Accuracy (%)	Sensitivity (%)	F1 Score (%)	Precision (%)
EEG δ band data					
Shannon entropy	1	82.35 ± 0.0	84.68 ± 2.89	90.32 ± 0.0	100 ± 0.0
	2	88.56 ± 0.42	88.65 ± 0.33	93.91 ± 0.21	100 ± 0.0
	3	88.36 ± 1.01	90.34 ± 0.34	93.68 ± 0.56	98.37 ± 0.90
	4	59.66 ± 0.88	60.66 ± 1.51	74.37 ± 0.62	99 ± 1.16
	5	82.08 ± 0.30	84.49 ± 1.71	90.14 ± 0.17	100 ± 0.0
Log energy entropy	1	82.35 ± 0.0	82.35 ± 0.0	90.32 ± 0.0	100 ± 0.0
	2	88.23 ± 0.0	88.23 ± 0.23	93.75 ± 0.0	100 ± 0.0
	3	88.35 ± 0.79	90.04 ± 0.33	93.69 ± 0.42	98.46 ± 0.67
	4	60.60 ± 1.08	61.23 ± 1.19	74.77 ± 0.61	99.28 ± 0.74
	5	82.20 ± 0.0	82.20 ± 0.0	90.23 ± 0.0	100 ± 0.0
SVD	1	82.50 ± 0.32	83.04 ± 1.34	90.39 ± 0.15	100 ± 0.0
	2	88.31 ± 0.26	88.31 ± 0.23	93.79 ± 0.13	100 ± 0.0
	3	88.13 ± 0.9	90.05 ± 0.37	93.58 ± 0.51	98.56 ± 1.20
	4	60.48 ± 1.41	62.11 ± 2.06	74.41 ± 0.54	97.87 ± 0.92
	5	82.20 ± 0.0	82.20 ± 0.0	90.23 ± 0.0	100 ± 0.0
EEG θ band data					
Shannon entropy	1	82.29 ± 0.07	85.61 ± 1.56	90.27 ± 0.04	100 ± 0.0
	2	87.52 ± 0.65	89.42 ± 0.63	93.30 ± 0.37	98.5 ± 0.926
	3	88.23 ± 0.0	88.78 ± 1.0	93.75 ± 0.0	100 ± 0.0
	4	47.16 ± 0.99	82.95 ± 7.89	27.25 ± 2.75	17.16 ± 2.09
	5	82.35 ± 0.96	84.30 ± 0.51	90.05 ± 0.53	97.73 ± 0.65
Log energy entropy	1	82.35 ± 0.0	82.35 ± 0.0	90.32 ± 0.0	100 ± 0.0
	2	88.79 ± 0.68	89.92 ± 0.40	93.94 ± 0.37	98.66 ± 0.66
	3	88.23 ± 0.0	88.23 ± 0.0	93.75 ± 0.0	100 ± 0.0
	4	59.49 ± 1.42	61.68 ± 0.89	72.93 ± 0.88	92.05 ± 1.65
	5	81.91 ± 0.70	84.19 ± 0.49	89.80 ± 0.46	97.93 ± 0.68
SVD	1	82.35 ± 0.0	82.45 ± 0.31	90.32 ± 0.0	100 ± 0.0
	2	87.53 ± 1.12	90.13 ± 0.56	93.22 ± 0.62	97.51 ± 0.92
	3	88.23 ± 0.0	88.23 ± 0.0	93.75 ± 0.0	100 ± 0.0
	4	52.70 ± 3.03	69.42 ± 3.87	52.44 ± 7.13	46.10 ± 10.58
	5	82.23 ± 1.44	84.38 ± 0.62	89.92 ± 0.85	97.01 ± 1.41
EEG α band data					
Shannon entropy	1	75.65 ± 5.32	86.73 ± 2.09	85.84 ± 3.62	90.68 ± 7.32
	2	88.31 ± 0.26	88.68 ± 0.62	93.78 ± 0.11	100 ± 0.0
	3	88.10 ± 1.33	89.50 ± 1.06	93.62 ± 0.69	99.23 ± 0.87
	4	63.34 ± 1.39	62.74 ± 0.67	75.44 ± 1.16	95.88 ± 1.56
	5	82.67 ± 0.43	82.91 ± 0.19	90.43 ± 0.23	100 ± 0.0
Log energy entropy	1	82.35 ± 0.0	82.35 ± 0.0	90.32 ± 0.0	100 ± 0.0
	2	88.46 ± 0.37	88.49 ± 0.34	93.86 ± 0.18	100 ± 0.0
	3	88.23 ± 0.0	88.30 ± 0.20	93.75 ± 0.0	100 ± 0.0
	4	61.35 ± 1.35	61.85 ± 0.60	74.24 ± 0.97	94.75 ± 1.34
	5	82.20 ± 0.0	82.20 ± 0.0	90.23 ± 0.0	100 ± 0.0
SVD	1	83.06 ± 0.25	83.17 ± 0.31	90.65 ± 0.12	100 ± 0.0
	2	88.23 ± 0.0	88.23 ± 0.0	93.75 ± 0.0	100 ± 0.0
	3	88.31 ± 0.23	88.70 ± 0.37	93.78 ± 0.11	100 ± 0.0
	4	61.68 ± 0.83	61.81 ± 0.56	74.37 ± 0.59	93.92 ± 1.49
	5	82.84 ± 0.33	82.85 ± 0.28	90.51 ± 0.19	100 ± 0.0
EEG SMR band data					
Shannon entropy	1	82.20 ± 0.25	87.01 ± 1.44	90.23 ± 0.15	99.89 ± 0.32
	2	87.25 ± 1.20	90.38 ± 1.19	93.13 ± 0.69	97.99 ± 1.38
	3	88.47 ± 0.55	89.26 ± 0.82	93.86 ± 0.29	99.90 ± 0.30
	4	62.35 ± 1.51	62.14 ± 0.88	74.92 ± 1.15	96.45 ± 1.68
	5	75.78 ± 9.05	91.55 ± 1.88	85.31 ± 7.32	89.89 ± 11.41
Log energy entropy	1	82.35 ± 0.0	82.35 ± 0.0	90.32 ± 0.0	100 ± 0.0
	2	88.55 ± 0.41	88.57 ± 0.35	93.90 ± 0.20	100 ± 0.0
	3	88.23 ± 0.0	88.23 ± 0.0	93.75 ± 0.0	100 ± 0.0
	4	61.58 ± 1.41	62 ± 1.04	74.35 ± 0.69	94.19 ± 2.04
	5	82.20 ± 0.0	82.20 ± 0.0	90.23 ± 0.0	100 ± 0.0
SVD	1	82.35 ± 0.0	82.35 ± 0.0	90.32 ± 0.0	100 ± 0.0
	2	89.28 ± 0.37	89.23 ± 0.36	94.26 ± 0.19	100 ± 0.0
	3	88.21 ± 0.40	88.52 ± 0.17	93.73 ± 0.02	100 ± 0.0
	4	62.33 ± 1.77	62.62 ± 0.94	74.46 ± 1.30	94.17 ± 1.40
	5	82.38 ± 0.38	82.78 ± 0.89	90.31 ± 0.17	100 ± 0.0
EEG β band data					
Shannon entropy	1	80.99 ± 1.36	86.90 ± 2.39	89.43 ± 0.81	98.04 ± 1.77
	2	88.38 ± 1.03	89.96 ± 0.31	93.73 ± 0.56	98.85 ± 0.87
	3	88.40 ± 0.35	91.26 ± 1.10	93.82 ± 0.15	100 ± 0.0
	4	52.02 ± 1.95	93.94 ± 6.89	36.80 ± 3.74	23.82 ± 3.04
	5	81.89 ± 0.87	83.83 ± 0.41	89.83 ± 0.54	97.52 ± 1.62
Log energy entropy	1	82.35 ± 0.0	82.35 ± 0.0	90.32 ± 0.0	100 ± 0.0
	2	88.19 ± 1.38	90.52 ± 0.46	93.55 ± 0.79	97.42 ± 1.42
	3	88.23 ± 0.0	88.23 ± 0.0	93.75 ± 0.0	100 ± 0.0
	4	59.61 ± 2.66	72.91 ± 3.25	63.17 ± 2.02	61.03 ± 3.21
	5	82.02 ± 1.46	83.76 ± 0.62	89.92 ± 0.87	98.14 ± 1.26
SVD	1	82.35 ± 0.0	82.35 ± 0.0	90.32 ± 0.0	100 ± 0.0
	2	87.40 ± 0.92	90.52 ± 0.2	93.11 ± 0.51	96.74 ± 1.02
	3	88.23 ± 0.0	89.92 ± 1.03	93.75 ± 0.0	100 ± 0.0
	4	55.62 ± 2.60	85.08 ± 5.36	47.92 ± 5.13	35.50 ± 5.63
	5	81.51 ± 1.48	84.19 ± 0.44	89.53 ± 0.86	96.80 ± 1.57
EEG γ band data					
Shannon entropy	1	82.60 ± 1.13	84.57 ± 0.44	90.15 ± 0.65	97.23 ± 0.83
	2	48.90 ± 5.42	94.09 ± 1.19	61.84 ± 5.86	47.51 ± 6.95
	3	88.62 ± 0.90	89.12 ± 0.36	93.90 ± 0.50	99.33 ± 0.90
	4	64.92 ± 0.98	63.95 ± 0.73	76.52 ± 0.64	97.72 ± 1.19
	5	37.85 ± 1.89	99.64 ± 1.12	40.32 ± 2.09	25.87 ± 1.41
Log energy entropy	1	83.23 ± 1.39	85.17 ± 0.66	90.55 ± 0.77	97.84 ± 0.75
	2	88.23 ± 0.0	88.23 ± 0.0	93.75 ± 0.0	100 ± 0.0
	3	88.23 ± 0.0	88.23 ± 0.0	93.75 ± 0.0	100 ± 0.0
	4	64.92 ± 1.66	64.27 ± 1.06	76.15 ± 1.02	95.46 ± 1.47
	5	82.36 ± 1.22	84.03 ± 0.99	90.08 ± 0.68	98.76 ± 1.06
SVD	1	82.43 ± 2.32	84.71 ± 1.05	90.05 ± 1.34	97.12 ± 1.92
	2	86.05 ± 2.49	90.10 ± 0.70	92.31 ± 1.44	95.40 ± 2.20
	3	89.04 ± 0.04	88.95 ± 0.04	94.15 ± 0.02	100 ± 0.0
	4	64.89 ± 1.56	63.87 ± 0.96	76.19 ± 0.88	95.61 ± 1.24
	5	45.20 ± 1.36	94.30 ± 2.83	52 ± 2.35	36.70 ± 2.43

Note—Class 1: schizophrenia; Class 2: mild cognitive impairment (MCI); Class 3: depression; Class 4: controls (healthy individuals); and Class 5: Alzheimer’s disease.

**Table 4:** SVM classification rates utilizing different EEG frequency band features.

Feature	Class Number	Accuracy (%)	Sensitivity (%)	F1 Score (%)	Precision (%)
EEG δ band data					
Shannon entropy	1	82.35	82.35	90.32	100
	2	88.23	88.23	93.75	100
	3	88.23	88.23	93.75	100
	4	59.66	59.66	74.73	100
	5	82.20	82.20	90.23	100
Log energy entropy	1	82.35	82.35	90.32	100
	2	88.23	88.23	93.75	100
	3	88.23	88.23	93.75	100
	4	59.91	59.81	74.85	100
	5	82.20	82.20	90.23	100
SVD	1	82.35	82.35	90.32	100
	2	88.23	88.23	93.75	100
	3	88.23	88.23	93.75	100
	4	59.66	59.66	74.73	100
	5	82.20	82.20	90.23	100
EEG θ band data					
Shannon entropy	1	82.35	82.35	90.32	100
	2	88.23	88.23	93.75	100
	3	88.23	88.23	93.75	100
	4	60.05	59.88	74.90	100
	5	82.20	82.20	90.23	100
Log energy entropy	1	82.35	82.35	90.32	100
	2	88.23	88.23	93.75	100
	3	88.23	88.23	93.75	100
	4	59.66	59.66	74.73	100
	5	82.20	82.20	90.23	100
SVD	1	82.35	82.35	90.32	100
	2	88.23	88.23	93.75	100
	3	88.23	88.23	93.75	100
	4	59.71	59.69	74.74	100
	5	82.20	82.20	90.23	100
EEG α band data					
Shannon entropy	1	82.35	82.35	90.32	100
	2	88.23	88.23	93.75	100
	3	88.23	88.23	93.75	100
	4	61.14	60.56	75.39	100
	5	82.20	82.20	90.23	100
Log energy entropy	1	82.35	82.35	90.32	100
	2	88.23	88.23	93.75	100
	3	88.23	88.23	93.75	100
	4	60.96	60.41	75.30	100
	5	82.20	82.20	90.23	100
SVD	1	82.35	82.35	90.32	100
	2	88.23	88.23	93.75	100
	3	88.23	88.23	93.75	100
	4	61.31	60.58	75.45	100
	5	82.20	82.20	90.23	100
EEG SMR band data					
Shannon entropy	1	82.35	82.35	90.32	100
	2	88.23	88.23	93.75	100
	3	88.23	88.23	93.75	100
	4	59.66	59.66	74.73	100
	5	82.20	82.20	90.23	100
Log energy entropy	1	82.35	82.35	90.32	100
	2	88.23	88.23	93.75	100
	3	88.23	88.23	93.75	100
	4	59.88	59.78	74.82	100
	5	82.20	82.20	90.23	100
SVD	1	82.35	82.35	90.32	100
	2	88.23	88.23	93.75	100
	3	88.23	88.23	93.75	100
	4	59.66	59.66	74.73	100
	5	82.20	82.20	90.23	100
EEG β band data					
Shannon entropy	1	82.35	82.35	90.32	100
	2	88.23	88.23	93.75	100
	3	88.23	88.23	93.75	100
	4	60.30	59.96	74.97	100
	5	82.20	82.20	90.23	100
Log energy entropy	1	82.35	82.35	90.32	100
	2	88.23	88.23	93.75	100
	3	88.23	88.23	93.75	100
	4	60.26	60.01	74.99	100
	5	82.20	82.20	90.23	100
SVD	1	82.35	82.35	90.32	100
	2	88.23	88.23	93.75	100
	3	88.23	88.23	93.75	100
	4	60.40	60.06	75.05	100
	5	82.20	82.20	90.23	100
EEG γ band data					
Shannon entropy	1	82.35	82.35	90.32	100
	2	88.23	88.23	93.75	100
	3	88.39	88.37	93.82	100
	4	64.12	62.97	76.32	99
	5	82.20	82.20	90.23	100
Log energy entropy	1	82.35	82.35	90.32	100
	2	88.23	88.23	93.75	100
	3	88.23	88.23	93.75	100
	4	64.78	63.60	76.45	98.58
	5	82.20	82.20	90.23	100
SVD	1	82.35	82.35	90.32	100
	2	88.23	88.23	93.75	100
	3	88.23	88.23	93.75	100
	4	65.74	64.13	77.16	98.30
	5	82.20	82.20	90.23	100

Note—Class 1: schizophrenia; Class 2: mild cognitive impairment (MCI); Class 3: depression; Class 4: controls (healthy individuals); and Class 5: Alzheimer’s disease.

**Table 5:** AdaBoost performance across different EEG frequency band features.

Feature	Class Number	Accuracy (%)	Sensitivity (%)	F1 Score (%)	Precision (%)
EEG δ band data					
Shannon entropy	1	82.35	82.35	90.32	100
	2	88.23	88.23	93.75	100
	3	88.23	88.27	93.75	100
	4	61.58	61.18	75.29	99.43
	5	82.20	82.20	90.23	100
Log energy entropy	1	82.35	82.35	90.32	100
	2	88.23	88.23	93.75	100
	3	88.23	88.27	93.75	100
	4	62.50	62.36	75.01	96.31
	5	82.20	82.20	90.23	100
SVD	1	82.35	82.35	90.32	100
	2	88.23	88.23	93.75	100
	3	88.23	88.26	93.75	100
	4	60.43	60.48	74.85	99.57
	5	82.20	82.20	90.23	100
EEG θ band data					
Shannon entropy	1	82.35	82.35	90.32	100
	2	88.23	88.23	93.75	100
	3	88.23	88.23	93.75	100
	4	62.22	61.57	75.50	99.01
	5	82.48	82.41	90.35	100
Log energy entropy	1	82.59	82.78	90.43	100
	2	88.23	88.23	93.75	100
	3	88.23	88.23	93.75	100
	4	60.67	60.62	74.90	99.43
	5	82.38	82.34	90.31	100
SVD	1	82.35	82.35	90.32	100
	2	88.23	88.23	93.75	100
	3	88.23	88.23	93.75	100
	4	62.16	61.64	75.33	98.72
	5	82.54	82.48	90.40	100
EEG α band data					
Shannon entropy	1	82.35	82.35	90.32	100
	2	88.23	88.23	93.75	100
	3	88.23	88.23	93.75	100
	4	62.13	61.76	75.33	98.86
	5	82.20	82.20	90.23	100
Log energy entropy	1	82.35	82.35	90.32	100
	2	88.23	88.23	93.75	100
	3	88.31	88.36	93.79	100
	4	61.63	61.13	75.25	99.28
	5	82.20	82.20	90.23	100
SVD	1	82.35	82.35	90.32	100
	2	88.23	88.23	93.75	100
	3	88.23	88.28	93.75	100
	4	63.15	62.35	75.88	99.71
	5	82.20	82.20	90.23	100
EEG SMR band data					
Shannon entropy	1	82.35	82.35	90.32	100
	2	88.23	88.36	93.75	100
	3	88.23	88.23	93.75	100
	4	60.55	60.52	74.86	99.43
	5	82.20	82.20	90.23	100
Log energy entropy	1	82.35	82.35	90.32	100
	2	88.40	88.78	93.83	100
	3	88.23	88.23	93.75	100
	4	61.85	61.89	74.83	96.44
	5	82.21	82.20	90.23	100
SVD	1	82.35	82.35	90.32	100
	2	88.23	88.23	93.75	100
	3	88.23	88.23	93.75	100
	4	61.23	61	75.08	99.29
	5	82.20	82.20	90.23	100
EEG β band data					
Shannon entropy	1	82.35	82.35	90.32	100
	2	88.23	88.23	93.75	100
	3	88.23	88.23	93.75	100
	4	61.55	61.82	74.52	95.46
	5	82.20	82.20	90.23	100
Log energy entropy	1	82.52	82.49	90.40	100
	2	88.23	88.23	93.75	100
	3	88.23	88.23	93.75	100
	4	61.95	62.17	74.53	94.04
	5	82.38	82.59	90.31	100
SVD	1	82.35	82.35	90.32	100
	2	88.23	88.23	93.75	100
	3	88.23	88.23	93.75	100
	4	60.66	61.38	74.27	97.45
	5	82.21	82.25	90.23	100
EEG γ band data					
Shannon entropy	1	82.57	82.58	90.43	100
	2	88.31	88.31	93.79	100
	3	88.97	88.95	94.11	100
	4	64.35	64.17	75.64	94.33
	5	82.41	82.45	90.30	100
Log energy entropy	1	82.64	82.58	90.46	100
	2	88.23	88.23	93.75	100
	3	88.23	88.23	93.75	100
	4	62.83	63.20	74.75	93.74
	5	82.21	82.20	90.23	100
SVD	1	82.57	82.57	90.43	100
	2	88.23	88.23	93.75	100
	3	88.97	88.95	94.11	100
	4	64.83	64.14	76.13	96.31
	5	82.81	82.75	90.52	100

Note—Class 1: schizophrenia; Class 2: mild cognitive impairment (MCI); Class 3: depression; Class 4: controls (healthy individuals); and Class 5: Alzheimer’s disease.

**Table 6:** KNN classification rates utilizing a combination of features across different EEG frequency bands.

Class Number	Accuracy (%)	Sensitivity (%)	F1 Score (%)	Precision (%)
EEG δ band data				
1	82.42 ± 0.45	83.77 ± 0.40	90.20 ± 0.28	98.77 ± 0.80
2	88.38 ± 0.54	88.87 ± 0.09	93.80 ± 0.28	99.80 ± 0.40
3	88.29 ± 0.46	88.68 ± 0.26	93.76 ± 0.24	99.90 ± 0.30
4	60.11 ± 2.05	64.82 ± 1.35	69.11 ± 1.89	75.74 ± 2.83
5	83.19 ± 1.05	84.47 ± 0.59	90.53 ± 0.62	98.76 ± 0.94
EEG θ band data				
1	82.45 ± 1.14	83.72 ± 0.65	90.18 ± 0.65	98.25 ± 0.84
2	88.75 ± 0.38	89.32 ± 0.37	93.96 ± 0.21	99.52 ± 0.50
3	88.57 ± 0.43	88.65 ± 0.36	93.91 ± 0.21	100 ± 0.0
4	61.22 ± 2.99	66.10 ± 2.43	69.61 ± 2.35	77.81 ± 3.62
5	81.15 ± 0.84	83.07 ± 0.24	89.43 ± 0.52	97.83 ± 0.90
EEG α band data				
1	82.46 ± 1.17	84.36 ± 0.74	90.12 ± 0.66	97.95 ± 0.67
2	88.90 ± 0.77	89.32 ± 0.45	94.06 ± 0.40	100 ± 0.0
3	88.38 ± 0.36	88.54 ± 0.35	93.82 ± 0.18	100 ± 0.0
4	58.05 ± 1.34	63.16 ± 0.98	67.14 ± 1.25	73.57 ± 1.36
5	81.53 ± 0.68	82.60 ± 0.36	89.76 ± 0.39	98.96 ± 0.48
EEG SMR band data				
1	82.64 ± 1.30	83.93 ± 0.70	90.26 ± 0.72	98.26 ± 0.68
2	88.61 ± 0.42	88.84 ± 0.10	93.93 ± 0.21	100 ± 0.0
3	88.58 ± 0.43	89.05 ± 0.33	93.91 ± 0.21	99.90 ± 0.30
4	61.68 ± 1.69	65.70 ± 1.15	70.01 ± 1.43	76.59 ± 1.81
5	81.85 ± 0.69	83.01 ± 0.44	89.90 ± 0.39	98.65 ± 0.84
EEG β band data				
1	82.57 ± 0.95	84.54 ± 0.57	90.19 ± 0.51	97.84 ± 0.75
2	88.85 ± 0.55	89.21 ± 0.49	94.04 ± 0.27	99.90 ± 0.30
3	88.80 ± 0.55	89.04 ± 0.29	94.03 ± 0.28	100 ± 0.0
4	62.55 ± 2.57	67.16 ± 2.27	70.60 ± 1.50	76.92 ± 2.78
5	81.87 ± 0.88	82.95 ± 0.76	89.88 ± 0.43	98.55 ± 0.53
EEG γ band data				
1	83.46 ± 1.17	84.90 ± 0.84	90.66 ± 0.61	98.26 ± 0.49
2	88.39 ± 0.36	88.73 ± 0.27	93.82 ± 0.18	100 ± 0.0
3	89.04 ± 0.76	89.98 ± 0.37	94.13 ± 0.39	99.71 ± 0.46
4	67.28 ± 2.45	70.50 ± 1.65	74.24 ± 2.06	80.45 ± 3.39
5	83.47 ± 1.35	85.05 ± 0.77	90.62 ± 0.77	97.93 ± 0.84

**Table 7:** Naïve Bayes classification rates utilizing a combination of features derived from various EEG frequency bands.

Class Number	Accuracy (%)	Sensitivity (%)	F1 Score (%)	Precision (%)
EEG δ band data				
1	60.10 ± 8.7	89.91 ± 1.97	71.57 ± 8.24	63.10 ± 13.11
2	87.18 ± 1.29	89.94 ± 0.67	93.05 ± 0.72	97.42 ± 1.42
3	86.76 ± 1.12	89.79 ± 0.46	92.77 ± 0.64	96.74 ± 1.12
4	59.79 ± 1.16	63.72 ± 3.09	73.40 ± 0.78	94.46 ± 1.06
5	64.32 ± 11.24	86.39 ± 1.17	75.73 ± 9.64	71.85 ± 17.59
EEG θ band data				
1	49.71 ± 7.35	92.90 ± 1.97	58.37 ± 9.37	44.72 ± 12.6
2	88.05 ± 1.47	90.91 ± 0.50	93.44 ± 0.85	97.22 ± 1.14
3	81.97 ± 3.88	92.27 ± 1.21	89.70 ± 0.49	89.82 ± 5.61
4	47.59 ± 1.72	81.12 ± 7.57	30.75 ± 3.10	20.06 ± 2.70
5	81.74 ± 1.75	85.18 ± 0.56	89.57 ± 1.01	95.97 ± 1.49
EEG α band data				
1	49.78 ± 5.48	88.68 ± 1.44	60.04 ± 6.15	46.50 ± 7.86
2	86.43 ± 2.04	92.11 ± 1.82	92.56 ± 1.27	96.46 ± 3.08
3	63.72 ± 5.25	92.06 ± 1.14	76.58 ± 4.29	67.84 ± 6.52
4	61.83 ± 1.36	62.12 ± 0.53	74.28 ± 1.14	93.20 ± 0.38
5	82.01 ± 1.10	83.33 ± 0.74	89.99 ± 0.62	98.86 ± 1.02
EEG SMR band data				
1	73.89 ± 8.07	88.76 ± 0.92	83.95 ± 6.77	86.60 ± 12.77
2	78.80 ± 4.59	91.18 ± 0.71	87.88 ± 2.91	87.65 ± 4.89
3	87.93 ± 0.70	90.02 ± 1.94	93.55 ± 0.39	99.32 ± 0.79
4	61.99 ± 2.07	62.45 ± 1.45	74.19 ± 1.06	93.47 ± 1.22
5	50.20 ± 3.72	93.05 ± 3.0	59.68 ± 4.94	45.87 ± 6.17
EEG β band data				
1	61.05 ± 11.88	87.73 ± 1.5	72.55 ± 11.17	66.92 ± 19.18
2	84.24 ± 1.19	91.86 ± 0.91	91.15 ± 0.66	92.74 ± 1.36
3	54.13 ± 4.52	95.03 ± 1.57	67.01 ± 4.68	53.25 ± 5.88
4	53.04 ± 1.38	86.88 ± 6.69	42.47 ± 3.53	29.76 ± 3.83
5	81.06 ± 0.91	85.18 ± 0.96	89.14 ± 0.58	95.25 ± 1.89
EEG γ band data				
1	81.33 ± 1.80	85.40 ± 0.93	89.30 ± 1.03	94.97 ± 1.71
2	35.13 ± 2.09	97.69 ± 2.49	44.06 ± 2.40	29.01 ± 2.05
3	88 ± 0.49	89.34 ± 0.86	93.56 ± 0.28	98.94 ± 0.70
4	65.79 ± 0.94	64.37 ± 0.73	76.90 ± 0.58	96.31 ± 1.18
5	38.84 ± 1.74	98.71 ± 2.07	42.59 ± 2.36	27.83 ± 1.18

**Table 8:** SVM classification rates utilizing a combination of features across different EEG frequency bands.

Class Number	Accuracy (%)	Sensitivity (%)	F1 Score (%)	Precision (%)
EEG δ band data				
1	82.35	82.35	90.32	100
2	88.23	88.23	93.75	100
3	88.23	88.23	93.75	100
4	61.41	60.72	75.48	100
5	82.20	82.20	90.23	100
EEG θ band data				
1	82.35	82.35	90.32	100
2	88.23	88.23	93.75	100
3	88.23	88.23	93.75	100
4	59.95	59.95	74.58	99.01
5	82.20	82.20	90.23	100
EEG α band data				
1	82.35	82.35	90.32	100
2	88.23	88.23	93.75	100
3	88.23	88.23	93.75	100
4	60.69	60.27	75.18	100
5	82.20	82.20	90.23	100
EEG SMR band data				
1	82.35	82.35	90.32	100
2	88.23	88.23	93.75	100
3	88.23	88.23	93.75	100
4	60.77	60.32	75.16	99.85
5	82.20	82.20	90.23	100
EEG β band data				
1	82.35	82.35	90.32	100
2	88.23	88.23	93.75	100
3	88.23	88.23	93.75	100
4	60.28	59.99	74.98	100
5	82.20	82.20	90.23	100
EEG γ band data				
1	82.35	82.35	90.32	100
2	88.23	88.23	93.75	100
3	89.06	88.97	94.16	100
4	66.16	65.59	76.46	94.48
5	83.13	82.97	90.69	100

**Table 9:** AdaBoost classification rates utilizing a combination of features in different EEG frequency bands.

Class Number	Accuracy (%)	Sensitivity (%)	F1 Score (%)	Precision (%)
EEG δ band data				
1	82.35	82.35	90.32	100
2	88.23	88.23	93.75	100
3	88.23	88.25	93.75	100
4	62.16	62.37	74.66	95.15
5	82.20	82.20	90.23	100
EEG θ band data				
1	82.57	82.61	90.43	100
2	88.31	88.31	93.79	100
3	88.23	88.23	93.75	100
4	61.33	61.49	74.35	96.19
5	82.67	82.66	90.44	100
EEG α band data				
1	82.35	82.35	90.32	100
2	88.23	88.23	93.75	100
3	88.23	88.29	93.75	100
4	62.86	62.67	74.99	95.30
5	82.20	82.20	90.23	100
EEG SMR band data				
1	82.35	82.35	90.32	100
2	88.63	88.96	93.95	100
3	88.23	88.23	93.75	100
4	62.17	62.54	74.40	93.20
5	82.20	82.20	90.23	100
EEG β band data				
1	82.50	82.47	90.39	100
2	88.31	88.31	93.79	100
3	88.23	88.23	93.75	100
4	61.65	62.66	74.06	93.50
5	82.20	82.24	90.23	100
EEG γ band data				
1	82.82	82.83	90.55	100
2	88.23	88.23	93.75	100
3	89.05	88.96	94.15	100
4	64.08	64.29	75.09	91.77
5	82.92	82.87	90.56	100

**Table 10:** Comparison of related studies on the detection of cognitive and mental disorders.

Reference	Dataset Size	Method	Results
[16]	62 individuals with schizophrenia and 70 healthy individuals	Feature extraction: Calculation of STE and the use of the Relief algorithm for selecting distinguishing features, along with the classifiers gNB, LDA, KNN, SVM, and RF.	The best results for KNN achieved an accuracy of 92.96%
[15]	34 individuals with depression and 30 healthy individuals	Feature extraction: Statistical analysis, spectral analysis, wavelet analysis, and functional connectivity with feature selection and the use of classifiers LINSVM, RBFSVM, and RF.	The best results were achieved with RBFSVM, demonstrating an accuracy of 99% and the most significant difference in the delta band
[17]	11 schizophrenia patients and 20 healthy controls	Feature extraction: Seventeen linear and non-linear metrics. Classifiers: KNN, logistic regression, decision tree, RF, and SVM.	The best results: KNN with an accuracy of 87%
[18]	45 schizophrenia patients and 39 healthy controls	Feature extraction: Using MVAR model to convert data into frequency-domain features in the alpha band. Classifiers: KNN, SVM, Decision Tree, and 3D-CNN.	Best results: 3D-CNN with an accuracy of 98.47%
[58]	15 schizophrenia patients and 14 healthy controls	Feature extraction: Calculation of the weighted phase lag index based on phase correlation and feature analysis using Degree D, clustering coefficient, global efficiency, local efficiency, and betweenness centrality.	Meaningful differences in AUC values in the temporal lobe regions
[19]	19 Alzheimer’s patients and 20 healthy controls	Feature extraction: Using six functional connectivity measures: Pearson correlation, wavelet correlation, large squared correlation, phase synchronization, phase locking, and phase lag index with ST-GCN and T-CNN classifiers.	Accuracy of 92.3% for ST-GCN and 89% for T-CNN
[20]	13 mild cognitive impairment-Alzheimer’s patients and 20 healthy controls	Feature extraction: Calculation of weighted connectivity index in frequency bands and utilization of the minimum spanning tree algorithm with the following metrics: degree, leaf fraction, diameter, eccentricity, betweenness centrality, and tree hierarchy.	Significant statistical reduction in mean power in the alpha and beta bands, indicating a less integrated system with reduced efficiency in data transmission in patients
[24]	20 Alzheimer’s patients and 20 healthy controls	Feature extraction: Pearson correlation, spectral correlation, sub-band correlation, weighted delay index, weighted phase delay index, phase locking, mutual information, and domain overlap correlation using GNN, SVM, and CNN.	Accuracy of 92% for GNN
[59]	44 mild cognitive impairment patients, 50 Alzheimer’s patients, and 67 healthy controls	Recorded with MEG, extracting meta-bands and parameterizing them using Absorption power, dominance degree, topological matching, displacement rate, and band complexity.	Identification of 3 main meta-bands and significant differences between healthy and patient groups
[33]	EEG recordings of 230 participants, including 28 with major depression, 42 with schizophrenia, 65 with mild cognitive impairment and Alzheimer’s disease, and 95 controls	The quantum potential according to Bohmian mechanics, combined with dendrogram representation of data and p-adic numbers.	AUC: 0.9143 (control vs. Alzheimer’s disease)
[60]	EEG recordings of 166 participants, including 28 with major depression, 42 with schizophrenia, and 95 controls	Personal universal dendrographic hologram (DH) signature or personal block DH signature	AUC: 0.9908 (control vs. Schizophrenia)AUC: 0.9986 (control vs. depression)
Proposed Method	EEG recordings of 230 participants, including 28 with major depression, 42 with schizophrenia, 65 with mild cognitive impairment and Alzheimer’s disease, and 95 controls	Various EEG frequency bands, Granger causality, various kinds of local graph structures, KNN, Naïve Bayes, SVM, and AdaBoost.	The highest classification accuracy of 89.36% using KNN

Note—STE: symbolic transfer entropy, GNB: Gaussian Naïve Bayes, LDA: linear discriminant analysis, SVM: Support Vector Machine, RF: random forest, MVAR: multivariate autoregressive, ST-GCN: spatial–temporal graph convolutional neural network, T-CNN: temporal convolutional neural network, GNN: graph neural networks. For a comprehensive description of the acronyms utilized throughout this manuscript, please refer to the Appendix A.

## Data Availability

This article examined EEG signals of the Dryad dataset [33], freely available in the public domain. The codes will be available from the corresponding author upon logical request.

## Appendix A. Table of Acronyms

**Acronym**	**Full Name**	**Description**
3D-CNN	Three-Dimensional Convolutional Neural Network	A neural network model that operates on 3D data structures, commonly used for analyzing video or volumetric data.
AdaBoost	Adaptive Boosting	An ensemble learning method that combines weak classifiers to create a strong classifier.
AUC	Area Under the Curve	A measure of the ability of a classifier to distinguish between positive and negative classes, derived from the ROC curve.
CNN	Convolutional Neural Network	A deep learning model particularly effective for processing image data by recognizing spatial hierarchies.
DSM-IV	Diagnostic and Statistical Manual of Mental Disorders, Fourth Edition	A manual published by the American Psychiatric Association, used by clinicians for diagnosing mental disorders.
DSM-V	Diagnostic and Statistical Manual of Mental Disorders, 5th Edition	The fifth edition of the manual for diagnosing mental disorders, published in 2013.
EEG	Electroencephalogram	A non-invasive test that measures electrical activity in the brain.
GCN	Graph Convolutional Network	A neural network designed to work with graph-structured data.
GNB	Gaussian Naive Bayes	A probabilistic classifier based on applying Bayes’ theorem with a Gaussian distribution assumption.
GNN	Graph Neural Network	A neural network architecture designed for graph-based data, capturing relationships between nodes.
ICD-10	International Classification of Diseases, 10th Edition	A system used globally to categorize and code health conditions, including mental health disorders.
KNN	K-Nearest Neighbors	A machine learning algorithm used for classification based on proximity in feature space.
LDA	Linear Discriminant Analysis	A classification method that finds a linear combination of features that best separates two or more classes.
LELGS	Logically Extended Local Graph Structure	A locally extended graph structure that is used to model relationships within data, extending logical connections between nodes.
ECT	Electroconvulsive Therapy	A medical treatment involving electrical stimulation of the brain to treat severe mental health conditions, such as depression.
TMS	Transcranial Magnetic Stimulation	A non-invasive procedure that uses magnetic fields to stimulate nerve cells in the brain, often used to treat depression and other disorders.
LGS	Local Graph Structures	A method used to extract local features from a matrix.
LINSVM	Linear Support Vector Machine	A version of SVM focused on linear classification.
MCI	Mild Cognitive Impairment	A condition characterized by noticeable memory and cognitive decline greater than expected for age but not severe enough to be classified as dementia.
MDD	Major Depressive Disorder	A mood disorder characterized by persistent feelings of sadness and loss of interest
MEG	Magnetoencephalography	A technique for measuring the magnetic fields generated by neural activity in the brain.
MVAR	Multivariate Autoregressive Model	A statistical model used to describe the relationship between multiple time series variables.
OVA	One-Versus-All	A classification strategy used in multi-class classification problems.
QPMVS	Quantum Potential Mean and Variability Score	A metric based on quantum theory to measure brain connectivity.
RBFSVM	Radial Basis Function Support Vector Machine	A type of SVM that uses a Radial Basis Function (RBF) kernel for classification tasks.
RF	Random Forest	An ensemble method using multiple decision trees to improve classification accuracy.
ROC	Receiver Operating Characteristic	A graphical plot used to evaluate the performance of a binary classifier.
SLGS	Symmetric Local Graph Structure	A symmetric local graph structure that employs symmetry in the relationships between nodes for data analysis and modeling.
SMR	Sensorimotor Rhythm	A frequency band in EEG typically associated with motor and sensory processing (12–15 Hz).
STE	Symbolic Transfer Entropy	A method used to quantify the directional flow of information between two time series in symbolic form. It is a measure used in the analysis of dynamic systems and has applications in neuroscience for studying brain connectivity.
ST-GCN	Spatial–Temporal Graph Convolutional Network	A deep learning model for analyzing brain data, considering spatial and temporal aspects.
SVD	Singular Value Decomposition	A matrix factorization method used to decompose a matrix into its singular values, which are useful for dimensionality reduction and feature extraction.
SVM	Support Vector Machine	A machine learning classifier that finds a hyperplane that best separates different classes in the feature space.
T-CNN	Temporal Convolutional Neural Network	A type of convolutional neural network designed for processing sequential or time-dependent data.
VLSG	Vertical Local Graph Structure	A vertical local graph structure typically used in hierarchical or relational data modeling, where nodes are connected in a vertical arrangement.
VSLGS	Vertical Symmetric Local Graph Structure	A vertical local graph structure that incorporates symmetry, applied in vertical relationships for more precise analysis.
ZHLGS	Zigzag Horizontal Local Graph Structure	A horizontally aligned local graph structure following a zigzag pattern, useful for modeling complex relationships with horizontal connections.
ZHMLGS	Zigzag Horizontal Middle Local Graph Structure	A zigzag horizontal local graph structure that specifically focuses on the middle section of the data, using zigzag connections for analysis.
ZVLGS	Zigzag Vertical Local Graph Structure	A vertically oriented local graph structure following a zigzag pattern, used in modeling complex relationships.
ZVMLGS	Zigzag Vertical Middle Local Graph Structure	A zigzag vertical local graph structure focused on the middle section of the data, applying zigzag connections in a vertical arrangement for detailed analysis.
